# Using individual-based bioenergetic models to predict the aggregate effects of disturbance on populations: A case study with beaked whales and Navy sonar

**DOI:** 10.1371/journal.pone.0290819

**Published:** 2023-08-31

**Authors:** Vincent Hin, André M. de Roos, Kelly J. Benoit-Bird, Diane E. Claridge, Nancy DiMarzio, John W. Durban, Erin A. Falcone, Eiren K. Jacobson, Charlotte M. Jones-Todd, Enrico Pirotta, Gregory S. Schorr, Len Thomas, Stephanie Watwood, John Harwood

**Affiliations:** 1 Institute for Biodiversity and Ecosystem Dynamics, University of Amsterdam, Amsterdam, The Netherlands; 2 Wageningen Marine Research, IJmuiden, The Netherlands; 3 Santa Fe Institute, Santa Fe, New Mexico, United States of America; 4 Monterey Bay Aquarium Research Institute, Moss Landing, California, United States of America; 5 Bahamas Marine Mammal Research Organization, Abaco, Bahamas; 6 Naval Undersea Warfare Center, Newport, Rhode Island, United States of America; 7 SEA Inc., Aptos, California, United States of America; 8 Marine Ecology and Telemetry Research, Seabeck, Washington, United States of America; 9 Centre for Research into Ecological and Environmental Modelling, University of St Andrews, St Andrews, United Kingdom; 10 Department of Statistics, University of Auckland, Auckland, New Zealand; MARE – Marine and Environmental Sciences Centre, PORTUGAL

## Abstract

Anthropogenic activities can lead to changes in animal behavior. Predicting population consequences of these behavioral changes requires integrating short-term individual responses into models that forecast population dynamics across multiple generations. This is especially challenging for long-lived animals, because of the different time scales involved. Beaked whales are a group of deep-diving odontocete whales that respond behaviorally when exposed to military mid-frequency active sonar (MFAS), but the effect of these nonlethal responses on beaked whale populations is unknown. Population consequences of aggregate exposure to MFAS was assessed for two beaked whale populations that are regularly present on U.S. Navy training ranges where MFAS is frequently used. Our approach integrates a wide range of data sources, including telemetry data, information on spatial variation in habitat quality, passive acoustic data on the temporal pattern of sonar use and its relationship to beaked whale foraging activity, into an individual-based model with a dynamic bioenergetic module that governs individual life history. The predicted effect of disturbance from MFAS on population abundance ranged between population extinction to a slight increase in population abundance. These effects were driven by the interaction between the temporal pattern of MFAS use, baseline movement patterns, the spatial distribution of prey, the nature of beaked whale behavioral response to MFAS and the top-down impact of whale foraging on prey abundance. Based on these findings, we provide recommendations for monitoring of marine mammal populations and highlight key uncertainties to help guide future directions for assessing population impacts of nonlethal disturbance for these and other long-lived animals.

## Introduction

Population dynamics of long-lived organisms are primarily determined by stochastic and seasonal environmental variability that affect reproductive success and juvenile survival [1–3]. To cope with such variability, long-lived animals have evolved a suite of physiological, behavioral and life-history adaptations that prioritize survival and optimize reproductive success over the course of their lifetime [4–6]. Because of their capacity to cope with variable environments, exposure of long-lived animals to human-induced stressors will generally alter individual behavior and physiology before any lethal effects occur [7]. However, for many wildlife populations the consequences of these nonlethal effects are hard to quantify [7, 8].

Both natural and anthropogenic sources can lead to disturbance of individuals, where disturbance is defined as a deviation in an animal’s physiology or behavior from patterns occurring in the absence of predators or humans [9, 10]. Natural predators cause nonlethal disturbance by inducing prey behaviors that reduce the risk of predation at the cost of reduced energy intake (e.g., vigilance) or increased metabolic costs (e.g., avoidance behavior) [11]. For example, predators can affect the distribution and resource intake of their prey by creating a ‘landscape of fear’ [12, 13]. Similar antipredator behaviors are also displayed in response to anthropogenic disturbance events [9, 14]. Both anthropogenic and natural disturbances can therefore be regarded as non-consumptive effects or trait-mediated indirect effects [15, 16], which are known to have substantial impacts on animal populations that may cascade down to affect entire communities [17–19].

Beaked whales (family *Ziphiidae*) are a group of deep-diving odontocetes that have become a conservation priority because of their sensitivity to military mid-frequency active sonar (MFAS) [20–23]. The use of MFAS has been implicated in several mass stranding events involving various beaked whale species, particularly Cuvier’s beaked whale, *Ziphius cavirostris*, hereafter *Zc* [24–27]. Besides these lethal effects, sonar-induced changes in diving behavior might have nonlethal effects on individual health and energetic status [28]. Tagging studies of beaked whales have recorded behavioral responses to sonar that can lead to loss of foraging opportunities, including avoidance of the sonar source, cessation of foraging and prolonged interruption between deep foraging dives [29–33]. While the effect of a single exposure is likely small, multiple exposures could have a chronic effect on individual health, with consequences for individual vital rates [7, 10].

Concerns about the population-level consequences of nonlethal effects of MFAS exposure are especially relevant for beaked whale populations that live in or around Navy training ranges, where sonar is used on a regular basis. Within the U.S. Navy’s Southern California Anti-Submarine Warfare Range (SOAR) around San Clemente Island, CA, the U.S. Navy regularly employs MFAS in an area that is prime quality habitat for *Zc* [34–37], as indicated by the high density and high site fidelity of *Zc* [38–40]. At SOAR, MFAS exposure was related to longer time intervals between successive deep dives that are associated with foraging, suggesting that MFAS leads to foraging disruption in *Zc* [30]. A similar situation occurs at the Atlantic Undersea Test and Evaluation Center (AUTEC), which is a U.S. Navy training range in the Bahamas and used by a resident population of Blainville’s beaked whales (*Mesoplodon densirostris*, hereafter *Md*) [41]. Joyce et al. [31] studied the movement and dive behaviors of *Md* at AUTEC during repeated, frequent and intense MFAS use as part of naval exercises and found clear and sustained displacement away from the core MFAS area in the majority of tracked whales [see also 42]. Dive behaviors during exposure were comparable to behaviors post- and pre-exposure, although the proportion of time spent at depths consistent with foraging decreased during initial exposure to MFAS [31]. Passive acoustic monitoring using bottom-mounted range hydrophones at AUTEC and at the Pacific Missile Range Facility (PMRF) in Hawaii also indicated that *Md* either leave the range, or reduce foraging vocalization upon exposure to sonar [33, 43–45], and that it may take 1–3 d post exposure before call rate returns to pre-exposure level [42, 46]. Taken together, these are strong indications that MFAS use disrupts the behavior of beaked whales, leading to ‘lost foraging dives,’ either through cessation of foraging or displacement away from preferred foraging areas.

Assessing the impact of federal activities on marine mammal populations is required by the United States’ Marine Mammal Protection Act, but translating short-term behavioral responses to population-level consequences has been difficult [7, 8]. The Population Consequences of Disturbance framework [10, 47] addresses this challenge by conceptually linking changes in individual behavior and physiology to changes in vital rates, potentially through chronic changes in individual health (broadly defined as all the internal factors that affect fitness or homeostasis). Modelling these processes requires the formulation of transfer functions that describe how behavior and physiology determine individual vital rates, potentially mediated through an effect on individual health. However, these transfer functions are complex and often of unknown nature, which makes it challenging to assess the potential impacts of environmental change or human-induced stressors on wildlife populations.

Part of the difficulty of predicting the population consequences of disturbance for long-lived animals stems from the challenge of scaling up short-term effects on individual behavior to population consequences that require the modelling of multiple generations. Exposure to the disturbance source may only last a few hours, but effects on vital rates may only become evident on a time scale of months or years (reproduction and offspring survival) or decades (adult survival). During this time, effects on health may be compensated or aggravated by other events. Although limited by small sample size, Joyce et al. [31] observed an increase in the proportion of time at depths consistent with foraging during the five days after *Md* were exposed to sonar at AUTEC. Indications of compensation were also found for *Zc* around SOAR [30]. Although whales might compensate for lost foraging by increasing foraging effort post exposure, the initial reduction in prey intake might still lead to a delayed initiation of reproduction or to reduced survival probabilities of dependent calves.

In this study, an individual-based model (IBM) was used to study how short-term, repeated, behavioral disturbances from MFAS at AUTEC and SOAR may affect beaked whale populations across multiple generations. Within this IBM, life histories of beaked whale individuals were governed by a dynamic bioenergetic model [48] and density-dependent population growth of whales emerged because foraging whales reduced prey availability. Telemetry data derived from satellite-tagged beaked whales were used to describe movement patterns of simulated whales around Navy training ranges and exposure to MFAS. Passive-acoustic monitoring data from range hydrophones were used to quantify the impact of MFAS on beaked whale foraging, and to create realistic time series of MFAS that were used as the disturbance source in the IBM. Lastly, recent insights into the distribution and abundance of beaked whale prey around Navy training ranges were used to represent spatial variation in habitat quality.

## Model

### Model overview

**Fig 1:**
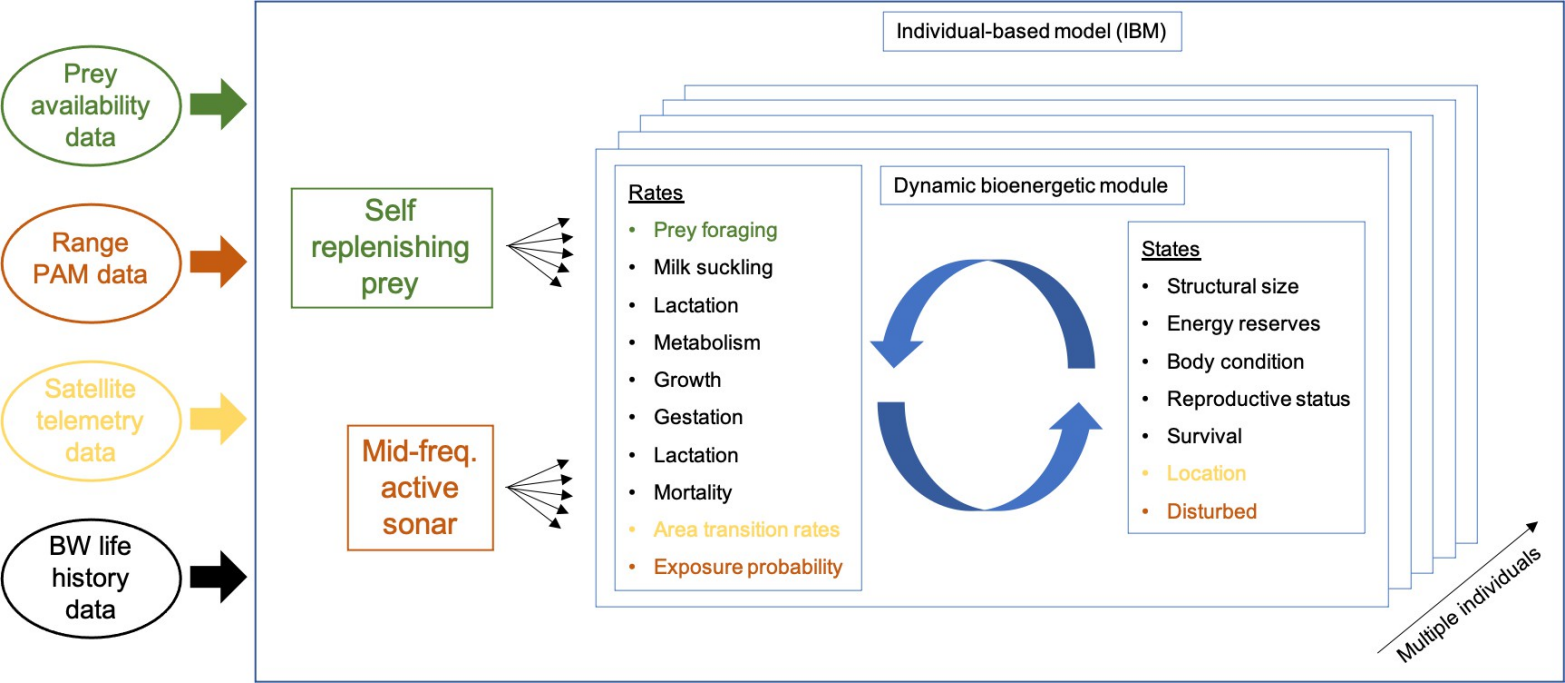
Different components of the IBM and their relationship to data used to estimate model components and/or processes. The IBM is comprised of a self-replenishing prey base and a module that simulates mid-frequency active sonar (MFAS). Each individual in the IBM is modelled using a dynamic bioenergetic module that calculates the progression of individual states based on rates that control energy intake, expenditure and movement between areas. Colors indicate the relationship between model components and their data counterparts.

We present an IBM to simulate the behavior (foraging and movement in response to MFAS), energetics and life history of *Zc* around SOAR and *Md* around AUTEC. Simulated whales were assumed to inhabit and move between discrete geographical areas (spatial units) that differed in habitat quality and MFAS use. Movement was modelled as a continuous-time Markovian process with transition rates derived from satellite telemetry data of tagged *Zc* and *Md* at SOAR and AUTEC, respectively [31, 42, 49] (Fig S2 in S1 File). To model the effect of behavior on energetics and life history, a general energy-budget model [50] for mammalian species previously applied to the long-finned pilot whale (*Globicephala melas*) [51, 52] was reparametrized for *Zc* and *Md*. In this model, beaked whale life history emerged from the rules that governed allocation of acquired energy from feeding on prey and suckling milk (for calves) to metabolic maintenance, growth in body size, gestation (for pregnant females) and lactation (for females with calves). An overview of the different model components and their relation to various input data is given in Fig 1. A detailed model description is available as supporting information (S1 File).

We considered a single, self-replenishing theoretical prey base across all areas in each location (SOAR and AUTEC). Area-specific differences in habitat quality were reflected in the catchability of prey (parameterized as prey encounter rate). Consequently, we assumed that prey productivity was linked between areas and foraging whales depleted prey across all areas, although the prey encounter rate was larger in high-quality areas, reflecting higher spatial aggregation of prey in these areas [53]. Top-down prey depletion by the whale population suppressed whale vital rates through the energy-budget model [51]. Negative density-dependent population growth therefore emerged from the interaction between whale foraging and prey productivity, as opposed to being explicitly modelled as a direct effect of whale density on reproduction or survival [54]. Because of the predator-prey coupling, an increase in overall prey productivity increased the modeled whale population density, as opposed to prey density, which was top-down controlled. In the absence of abundance estimates from unexposed beaked whale populations, we used prey productivity values that resulted in undisturbed stationary whale population with abundances close to, or slightly higher than the estimated abundances of *Zc* around SOAR (approximately 100 individuals) and *Md* around AUTEC (approximately 40 individuals) [41, 55, 56].

### Navy ranges

SOAR is located off the coast of California, west of San Clemente Island, and is part of the broader U.S. Navy Southern California Range Complex [30, 57]. The range is approximately 1,800 km^2^ and instrumented with an array of 177 bottom-mounted hydrophones used for Navy training purposes [57]. Although Navy exercises involving MFAS are concentrated on the range, MFAS exposure can occur throughout the entire range complex [30]. The range and surrounding area supports a density of *Zc* that is considered high for this species [37]. *Zc* individuals are regularly detected both acoustically and visually on SOAR and display a high site fidelity to the area [30, 35, 49].

AUTEC is located east of Andros Island, the Bahamas, in the Tongue of the Ocean, which is part of the Great Bahama Canyon, a deep-water trench that extends south from the Northwest Providence Channel [41]. This range is approximately 1,500 km^2^ and equipped with 91 bottom-mounted hydrophones [44, 46]. The resident population of *Md* at AUTEC has one of the highest densities of *Md* that has ever been estimated [37, 58, 59] and is subject to a long-term photo-identification program [41]. In the following, we use the term ‘range’ to refer to the hydrophone arrays on SOAR or AUTEC.

### Division of areas

For each location (SOAR and AUTEC) the (number of) modeled areas (spatial units) were defined based on MFAS use and available information on habitat quality (defined in section “habitat quality”). Areas were categorized as either high- or low-quality habitat to minimize the number of areas per location and ensure convergence of the estimation of transition rates (see *movement* section). Assessments of spatial variation in the quality of beaked whale habitat at SOAR and AUTEC are presented in [60] and [53], respectively. Specifically, both ranges were divided into a western, high-quality area and an eastern low-quality area. Because of the specific topography of the Tongue of the Ocean, we distinguished three off-range areas that collectively encompassed all locations of tagged individuals that were not on AUTEC (Fig S2a in File S1). Following [53], for each on-range area at AUTEC, there was one off-range area to the north that had the same habitat quality as its neighboring on-range area. In addition, we considered a single low-quality off-range area south of AUTEC [53]. At SOAR, we modelled a single off-range area that we assumed was free of sonar and of low quality (Fig S2b in File S1).

### Habitat quality

Following [60], habitat quality was quantified as the number of dives per day required to meet the basal metabolic need of a typical adult beaked whale, with high values indicating low habitat quality. This metric combined data on prey fields, prey energetic content and prey spatial aggregation with estimates of swimming speed and metabolic rate of adult beaked whales. As our model only accounted for a single prey base, differences in habitat quality were reflected in the area-specific prey encounter rates (attack rates). Without loss of generality, we set the prey encounter rate for low quality habitats to one and used the ratio of the number of dives required in low-quality habitat to the number required in high-quality habitat ($\phi_{R_{high}}$) as an estimate for the prey encounter rate in high-quality areas. For SOAR, the mode for the number of dives per day required to meet the baseline daily metabolic requirement of an adult *Zc* was 1.5 for high-quality habitat and 20 for low-quality (Table 1 in [60]). For *Zc* at SOAR, we therefore adopted $\phi_{R_{high}}=\frac{20}{1.5}=13.3$. For *Md* at AUTEC, the dive rate required to meet metabolic demands was 1.2 and 6.0 for high- and low-quality habitats, respectively, [53] and we therefore adopted $\phi_{R_{high}}=\frac{6.0}{1.2}=5.0$. To account for uncertainty in the prey data underlying these default estimates, we varied $\phi_{R_{high}}$ upon analysis.

### Movement

Modelled whales could freely transit between areas and transition rates were derived from satellite telemetry data of *Md* tagged within or near AUTEC (*n* = 7) and *Zc* tagged around SOAR (*n* = 12; Fig S2 in File S1). Details of tag deployment and data collection are described in [31] for *Md* and [30] for *Zc*. Tags on *Zc* were deployed under the US National Marine Fisheries Service permit numbers 540–1811 and 16111 in accordance with the Institutional Animal Care and Use Committee guidelines for satellite tagging established by Cascadia Research Collective. Tagging of *Md* was conducted under a Bahamas Marine Mammal Research Permit (permit #12A), issued by the Government of the Bahamas to the Bahamas Marine Mammal Research Organisation under the regulatory framework of the Bahamas Marine Mammal Protection Act (2005). Methods of deployment, tag types, and sample sizes were preapproved by the Institutional Animal Care and Use Committee of the Bahamas Marine Mammal Research Organisation and by the US Department of the Navy, Bureau of Medicine and Surgery Veterinary Affairs Office.

The estimation procedure for fitting transition rates is described in [42]. In short, the raw Argos telemetry data were filtered to exclude highly unrealistic animal locations, after which a continuous-time correlated random walk state-space model was used to adjust filtered tracks for Argos uncertainty [42]. Transition rates between areas were quantified by fitting a discrete-space, continuous-time Markov model on these filtered, adjusted locations. This procedure resulted in a continuous-time transition rate matrix **Q**, each element *q*_*i*,*j*_ of which described the immediate risk of moving from area *i* (rows) to area *j* (columns). We did not consider time-dependence of transition rates or dependence on any covariates. If sonar exposure led to displacement from the range, modelled individuals were translocated to an off-range area (see *disturbance* section), but their subsequent transition rates remained unaffected. Therefore, we assumed that the fitted transition rates reflected baseline movement patterns of *Md* and *Zc* around their respective ranges. Calves were assumed to have the same location as their mothers at all times.

### Energetics

Details of the energy budget model and derivation of its parameters are presented in the S1 File. The model was based on physiological principles of long-lived mammals, assuming that growth and reproduction proceeded at rates independent of current food conditions while individuals adapted their foraging rate to maintain a target body condition [50]. A number of rules (i.e., functions) described how the state of the individual influenced its survival and the allocation of energy towards growth and reproduction (gestation and lactation). We distinguished reserve mass (metabolizable energy stores) from structural mass (non-metabolizable compounds). Further, whales were characterized by their age, sex, structural length and total body mass (sum of structure and reserves). Structural length was directly related to age according to a Von Bertalanffy function. A length-weight relationship was used to calculate structural mass from structural length. We assumed no sex-specific differences in structural size and an equal sex ratio at birth.

Either the absolute amount of reserves or its relative extent (i.e., the ratio between reserve mass and total body mass, referred to as *body condition*) determined processes such as feeding, lactation, mortality and initiation of reproduction. Individual body condition determined prey feeding rate and milk consumption rate (for calves) through a so-called “feeding level” (0–1), which was a decreasing, sigmoidal function of body condition. The feeding level was higher for whales in poor body condition, which could result from low prey availability, high energy expenditure (e.g., during lactation) or disturbance. In contrast, the feeding level was assumed to decrease at high body condition, ensuring that reserve mass did not grow out of bounds under favorable conditions. For lactating females, individual body condition affected the milk provisioning rate. Females in poor condition reduced milk supply to their calf and ceased supply completely when their body condition dropped below a starvation threshold value. Age and structural size also influenced prey feeding rate and milk consumption rate for calves. Prey feeding increased with age throughout the early years of life (for calves and newly weaned individuals), while milk consumption rate declined with age up to the age at weaning, when lactation stopped. In addition, milk feeding rate was proportional to calf structural surface area [52]. Prey feeding followed a type II functional response in relation to prey density, with area-specific prey encounter rates (attack rates) and a maximum prey consumption rate that was proportional to structural surface area. All whales suffered from natural background mortality. Calves and weaned females experienced age-dependent background mortality according to a Siler model [61, 62], while weaned males have constant background mortality rate. In addition to natural mortality, whales with a body condition below the starvation threshold level suffered an additional risk of mortality that increased with declining body condition. Reproduction was initiated when absolute reserve mass exceeded a predetermined pregnancy threshold value, following [63].

Depending on an individual’s reproductive state, energy expenditure consisted of four different costs. For all individuals, field metabolic rate is three-quarter-power function of maintenance body mass, which was defined to account for the lower mass-specific metabolic costs of reserves relative to structure (see S1 File for details). For all individuals, growth costs were given by the change in structural mass multiplied by a cost-of-growth parameter. For fully-grown individuals, growth costs were negligible. The mass of the fetus was included in the maintenance body mass of pregnant females, and gestation costs were proportional to the structural growth of the fetus. For lactating females, lactation costs were proportional to milk consumption of their calf. Together with the rates of energy intake, the rates of energy expenditure determine reserve mass dynamics. Reserve mass increased when total energy intake exceeded total energy expenditure and decreased when the opposite was true. The metabolic efficiency of storing reserve mass (anabolism) differed from the efficiency of mobilizing reserve mass (catabolism).

### Exposure to MFAS

For both ranges, information on MFAS-use and beaked whale echolocation were derived from passive acoustic monitoring of range hydrophones. Data consisted of hourly counts of beaked whale dive starts detected on each hydrophone and hourly presence/absence of sonar detected on each hydrophone across two years (2013–2014 for AUTEC and 2014–2015 for SOAR) [30, 46, 57, 64]. Data gaps, during which no monitoring occurred, were excluded. This resulted in 16,400 hours of data for AUTEC (1.87 years) and 11,925 hours for SOAR (1.36 years). Hydrophones were assigned to their spatial unit within each range (western vs eastern spatial units) and the area covered by each hydrophone was calculated using a tessellation algorithm (see [43] for details). Within each spatial unit, the areas associated with hydrophones on which sonar was detected were summed and scaled by the maximum area over which sonar was detected across the two years for each range. This resulted in a standardized measure of sonar use, which we refer to as the ‘standardized sonar area’. Hourly detections of beaked whale dive starts were summed across all hydrophones within each spatial unit. We fitted separate Generalized Additive Models (GAMs) for each range to model the number of dive starts per spatial unit per hour as a function of a series of covariates. Specifically, each GAM tested for the effects of 1) standardized sonar area; 2) spatial unit within the range (western vs eastern areas); 3) year; 4) hour (time of day); 5) Julian day; 6) time since onset of sonar and 7) duration of the sonar bout. The response variable was assumed to follow a Poisson distribution, with a log-link function and the size of the spatial unit as an offset on the link scale. Continuous covariates were modelled using thin-plate splines, except for hour and Julian day, which were cyclic cubic regression splines so that the estimated effect at the beginning and end of the day or year matched. Smoothness was estimated by minimizing an unbiased risk estimator (UBRE) criterion. Analysis of hydrophone data was done with the ‘mgcv’ package in R [65, 66].

After back-transformation to the original scale, the predicted effect of standardized sonar area on the beaked whale dive-start rate provided an estimate of the relative decrease in beaked whale foraging activity (*z*) as a result of sonar use. Here, *z* = 1 indicated no reduction in beaked whale foraging in a particular area, while *z* = 0 indicated a complete reduction of foraging. For each hour, we calculated values of *z* specific for each spatial unit based on the standardized sonar area within that hour. This resulted in two time series of MFAS use per location: *z*_*w*_ and *z*_*e*_ for the western and eastern spatial units, respectively. These time series were condensed by grouping consecutive hours of identical *z*-values into sonar events using run-length encoding, such that each event was characterized by a starting time (d), a *z*-value and a duration (in multiples of one hour). For each location, the time series of events for eastern and western spatial units served as input to the IBM. In model scenarios with sonar, we repeated the same time series of sonar use until the end of the model simulation. Because our main study objective was to explore potential population effect of MFAS use, we only used the mean effect of the standardized sonar area on beaked whale dive count rate in the IBM. The other terms were incorporated in the GAM as covariates.

### Disturbance

Whales were assumed to be exposed to sonar if they were present on a range area at the onset of a sonar event. Upon exposure, the *z*-value of that particular event was used to calculate the number of disturbed whales. We simulated three different behavioral responses to MFAS: 1) cessation of foraging for a certain period of time, 2) displacement to an off-range area and 3) both cessation of foraging and displacement. To clearly distinguish between effects of displacement and cessation of foraging, we assumed that displaced whales did not lose foraging time and continued foraging in the area they were displaced to. In the cessation-of-foraging scenario, we calculated the number of whales that ceased foraging as *N*_*i*,*d*_ = (1 –*z*_*i*_) *N*_*i*_, with *N*_*i*_ being the number of whales in a particular on-range area *i* at the onset of the sonar event (see *Division of spatial units* and Table 1 for all areas) and subscript *d* representing disturbed whales. Variable *z*_*i*_ refers to the beaked whale foraging activity in relation to MFAS use for area *i*. Whales already disturbed by a previous sonar event were included in *N*_*i*,*d*_, to ensure that the fraction of disturbed whales within each area (*N*_*i*,*d*_ */ N*_*i*_) matched the observed sonar-induced decrease in beaked whale foraging activity (*z*) as predicted by the GAM. A number of *N*_*i*,*d*_ whales were randomly selected amongst the previously undisturbed whales. For each selected whale, the prey foraging rate was set to zero and a disturbance duration (*t*_*d*_) was sampled. This duration was assumed to follow an Erlang distribution (a common waiting time distribution) with shape parameter *k* = 2 and scale parameter *μ* = 0.75, which resulted in a default mean disturbance duration of 1.5 days with a standard deviation of 1.06 days. The disturbance duration was used to set the future time at which the whale would resume foraging: *t + t*_*d*_. Although it is unlikely that whales off range will never be exposed to MFAS, for simplicity we assumed that simulated whales not present on the range at the onset of a sonar event could not be disturbed.

**Table 1:** Transition rate matrices and stable distributions of *Md* around AUTEC and *Zc* around SOAR.

	*Mesoplodon densirostris*	*Ziphius cavirostris*
Spatial units	[south, western range, northwest, northeast, eastern range]	[off range, western range, eastern range]
Transition rate matrix (**Q**)	$\left[\begin{array}{ccccc} -0.2724 & 0.2022 & 0 & 0 & 0.0702\\ 0.1964 & -0.8101 & 0.1676 & 0.0339 & 0.4122\\ 0 & 0.1911 & -0.6488 & 0.4464 & 0.0113\\ 0 & 0.0173 & 0.8364 & -0.9048 & 0.0511\\ 0.1816 & 1.6187 & 0 & 0.481 & -2.2813 \end{array}\right]$	$\left[\begin{array}{ccc} -0.1397 & 0.1077 & 0.032\\ 0.257 & -0.5077 & 0.2507\\ 0.1836 & 1.2906 & -1.4743 \end{array}\right]$
Stable distribution **Q**	[0.21, 0.24, 0.31, 0.19, 0.055]	[0.64, 0.30, 0.065]
Fraction on-range **Q**	0.29	0.36
Adjusted transition rate matrix (**Q**_adj_)	$\left[\begin{array}{ccccc} -0.2724 & 0.2022 & 0 & 0 & 0.0702\\ 0.01964 & -0.08101 & 0.01676 & 0.00339 & 0.04122\\ 0 & 0.1911 & -0.6488 & 0.4464 & 0.0113\\ 0 & 0.0173 & 0.8364 & -0.9048 & 0.0511\\ 0.01816 & 0.16187 & 0 & 0.0481 & -0.22813 \end{array}\right]$	$\left[\begin{array}{ccc} -0.1397 & 0.1077 & 0.032\\ 0.0257 & -0.05077 & 0.02507\\ 0.01836 & 0.12906 & -0.14743 \end{array}\right]$
Stable distribution **Q**_adj_	[0.057, 0.66, 0.084, 0.052, 0.15]	[0.15, 0.70, 0.15]
Fraction on-range **Q**_adj_	0.81	0.85

In the displacement scenario, the number of whales displaced was calculated relative to the stable distribution of individuals across areas. This distribution followed the baseline movement pattern as dictated by the transition rate matrix **Q**. Specifically, the number of whales displaced equaled: *N*_*i*,*d*_ *=* max(*N*_*i*_*−M*_*i*_ *z*_*i*_, 0). Here, *M*_*i*_ *= p*_*i*_ *N*_*tot*_ is the number of whales in area *i* according to the stable proportion *p*_*i*_, with $\sum_{i=1}^{n}p_{i}=1$, *n* being the number of areas and *N*_*tot*_ total population size. As for the cessation of foraging, this calculation ensured that the modelled decrease in foraging activity within an area matched its observed counterpart as predicted by the GAM fitted to the dive-start data. The randomly selected *N*_*i*,*d*_ whales were displaced to an off-range area, but their baseline transition rates remained unaffected. For *Md* at AUTEC, we assumed that whales displaced from the range moved to the off-range area directly to the north [31]. At SOAR there was only a single off-range area that whales could be displaced to. This same procedure was adopted in the scenario where MFAS led to both displacement and cessation of foraging, but, in addition, the prey foraging rates of displaced whales were set to zero for a randomly selected period drawn from the Erlang distribution described above.

### Model simulation

The Escalator Boxcar Train (EBT) software package (https://staff.fnwi.uva.nl/a.m.deroos/ EBT/Software/ index.html) [67] was used to simulate the IBM. This package contains numerical integration routines to compute the continuous-time dynamics of the individual-state variables (Table S1 in S1 File) of all individuals in the population, together with the ordinary differential equation that describes prey dynamics (Table S2 in S1 File). Model implementation files for use with EBT-software are available online (https://osf.io/2ftmj/). The movement of each individual between different geographical areas was simulated by calculating, for each area *j* other than the individual’s current area *i* (*i* ≠ *j*), the future time of moving to area *j* as a random draw from an exponential distribution with rate *q*_*i*,*j*_. The individual was assigned to move to the area with the earliest future time of moving.

We simulated the onset of MFAS use on a previously undisturbed population residing at its stationary state, distinguishing between the three different behavioral responses to MFAS. Because prey productivity rates were adjusted to match the observed small beaked whale population abundances, there were considerable effects of demographic stochasticity. Therefore, we ran 100 replicate simulations for each behavioral response scenario and reported the mean (+/- sd) population density. We inferred the rate of population decline from the dynamics of total population abundance. For the cessation-of-foraging scenario, we studied how fast the population would recover once use of MFAS stopped. Besides effects on population abundance, we studied effects on age at onset of reproduction and female body condition. In addition, we studied the effect of varying 1) the difference in habitat quality between low- and high-quality habitats (as quantified by $\phi_{R_{high}}$) and 2) the transition rate matrix **Q**. For each combination of these, we readjusted the parameter describing maximum prey density (*R*_*max*_) to ensure that undisturbed population abundances remained comparable. The sensitivity to the transition rate matrix **Q** was explored by applying a 10-fold decrease to all rates that described transitions from range areas to off-range areas. Further, we studied the effect of the mean disturbance duration and to isolate the contribution of MFAS use on the model outcomes, we applied the derived MFAS time series from AUTEC to *Zc* at SOAR and vice versa.

## Results

### Movement and stable distribution

The transition rate matrices estimated from beaked whale telemetry data indicated that, on average, *Zc* around SOAR spent seven days off range, two days in the western area of the range and less than one day (16 hours) in the eastern part of the range (Table 1). According to the associated stable distribution, 64% of *Zc* population at SOAR was off range, 30% was in the western area of the range and only 6.5% was in the eastern area. For *Md* at AUTEC, the movement analysis involved five different areas and there was less variation in mean residency times among areas compared to *Zc* at SOAR (Table 1). The longest residency time occurred in the area south of the range (mean of 3.7 days) and the shortest in the eastern range area (mean of 11 hours). The associated stable distribution (Table 1) indicated that on average 29% of the *Md* population could be found on AUTEC, the large majority (81%) of which were in the western area (24% vs 5.5%). The north-western off-range area hosted the largest percentage of the *Md* population (31%). The north-eastern and southern off-range areas hosted similar percentages of the population (19% and 21%, respectively).

### Exposure and response to MFAS

**Fig 2:**
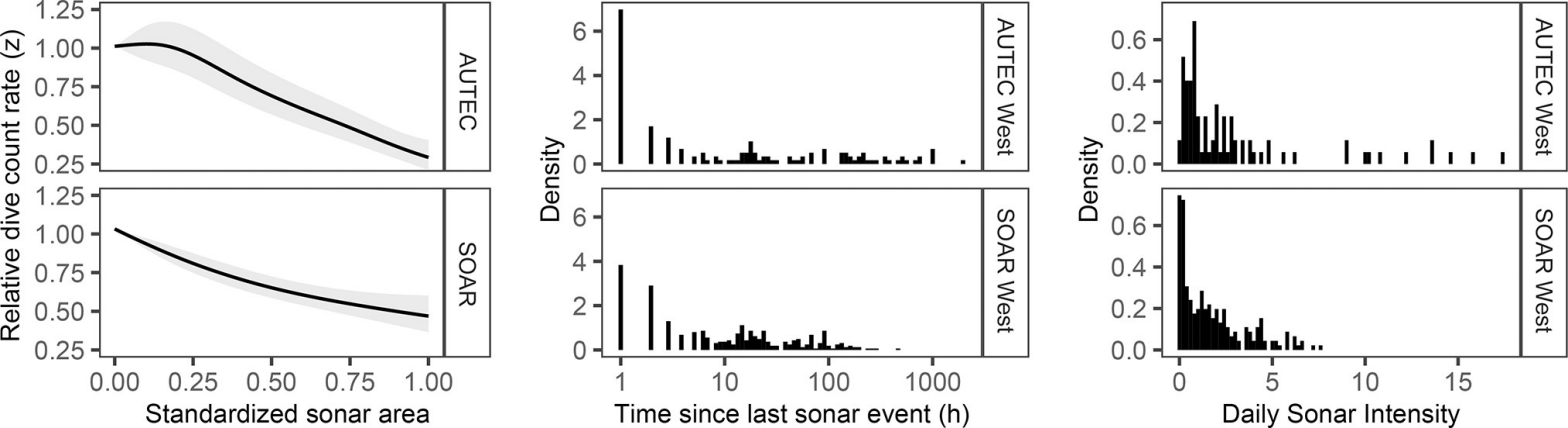
Characteristics of MFAS use on SOAR and AUTEC Navy training ranges. Left panel shows the GAM prediction of the effect of standardized sonar area on the relative dive count rate (number of beaked whale dive starts per hour). A relative dive-count rate of 1 means no decrease and a dive-count rate of 0.5 implies a 50% lower dive-count rate. Middle panels: the distributions of the time-interval between different sonar events on western range areas. Right panels: distributions of the daily sum of ‘standardized sonar area’ for western range areas. Corresponding plots for eastern range areas are highly similar (Figs S5 and S6 of S1 File).

For the two years of data analyzed, MFAS was used more frequently on SOAR than on AUTEC, with sonar events occurring in 8.4% and 2.9% of all recorded hours on each range. On AUTEC, there were longer time intervals between sonar events, but there were more days with high MFAS use (as quantified by the daily sum of the standardized sonar area; Fig 2). Mean standardized sonar area for all hours with sonar was higher for the western range areas on AUTEC than on SOAR (0.537 vs. 0.384, respectively), but this was not the case for the eastern range areas (0.411 vs 0.456). For both ranges, the hourly sonar count (number of hydrophones on which sonar was received) on the eastern and western areas were strongly correlated (Pearson correlation coefficient 0.848 for AUTEC and 0.863 for SOAR).

Standardized sonar area had a negative effect on the number of dive starts per hour, a measure of beaked whale foraging activity (Fig 2). On AUTEC, there was little effect on beaked whale dive count rate at low sonar intensity, but the relative decrease in dive count rate on AUTEC at the maximum recorded MFAS use was larger than on SOAR (0.71 vs 0.53; Fig 2). For AUTEC, the GAM revealed significant effects of ‘area’ and ‘year’, with higher dive-count rates in the western area and in 2013 (Table S4 of S1 File). There were fluctuations in dive-count rate throughout the year and day, and a weak pattern of increasing dive-count rate with increasing time since last MFAS use (Fig S3 in S1 File). For SOAR, the results from the GAM were similar (Table S5 in S1 File). Dive-count rate was higher in the western SOAR area and in 2014 (compared to 2015). There was also significant variation throughout the day and year, and a weak effect of time since last MFAS use (Fig S4 in S1 File). There was also an effect of the duration of the sonar bout, but this effect disappeared when temporal autocorrelation was accounted for (Table S5 and Fig S4 in S1 File).

### Population consequences of disturbance

**Fig 3:**
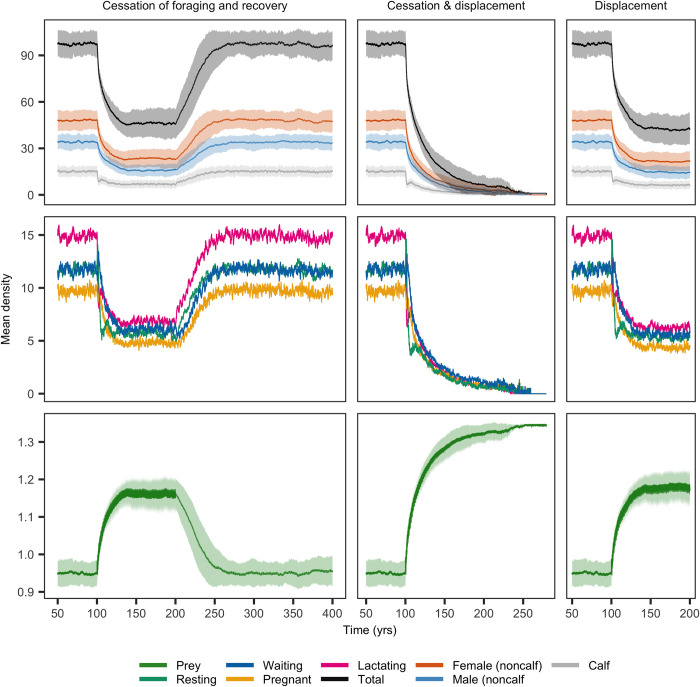
Population dynamics of *Zc* at SOAR in response to onset of MFAS disturbance at time *t* = 100 y. Disturbance either led to cessation of foraging (left panels), displacement from range (right panels) or both (center panels), in which case the *Zc* population went extinct. In the first scenario, the recovery of the population after cessation of MFAS disturbance at time *t* = 200 y is plotted. Trajectory of population recovery for the displacement response scenario was similar. Lines and shaded areas indicate mean and standard deviation of 100 replicate simulations, respectively. Middle rows show reproductive classes of females and standard deviations are omitted here for sake of presentation. Note the differences in time scales between panels. All model parameters at default values (Table S3 of S1 File).

The onset of MFAS use led to a rapid initial decline of *Zc* population abundance at SOAR and a subsequent slower approach of the population to a new stationary state (Fig 3). This decline occurred across all three behavioral response scenarios, but was greatest if MFAS led to both displacement and cessation of foraging, in which case the *Zc* population went extinct in all replicate simulations. Either displacement or cessation of foraging alone led to an approximately 50% reduction of population size. For the behavioral response scenario involving cessation of foraging, half of the decrease in population abundance occurred within the first 5 years after the onset of MFAS, but it took another 26 years before mean population abundance declined to within 1 per cent of the stationary population abundance under disturbance. Among females in the different reproductive classes, the decline in abundance was most pronounced for lactating females. Because of the coupling between predator and prey dynamics, prey density increased in response to the relaxation of foraging pressure caused by the decline in whale population abundance (Fig 3). The magnitude of the increase in prey density mirrored the reduction in abundance of the *Zc* population following the onset of MFAS use. Disturbance from MFAS also increased the temporal variation in mean prey density, with temporal peaks corresponding to disturbance-induced reduction of beaked whale foraging, followed by a decline in prey density in the intervals between MFAS events. The rate of recovery of the *Zc* population when MFAS ceased was slower than the initial population decline. On average, it took 66 years for the *Zc* population to recover from MFAS disturbance that led to cessation of foraging (Fig 3). During the first 30 years of recovery, the annual population growth rate was 1.13%. The trajectory of population recovery was equal between all scenarios, although the initial population abundance was higher for those scenarios in which the effect of MFAS was less. It should be emphasized that apart from the first 100 years and the recovery phase (Fig 3 left panels), these population trajectories represent long-term exposure simulations in which the use of MFAS continued throughout the decline and stabilization of the population to a new stationary state.

**Fig 4:**
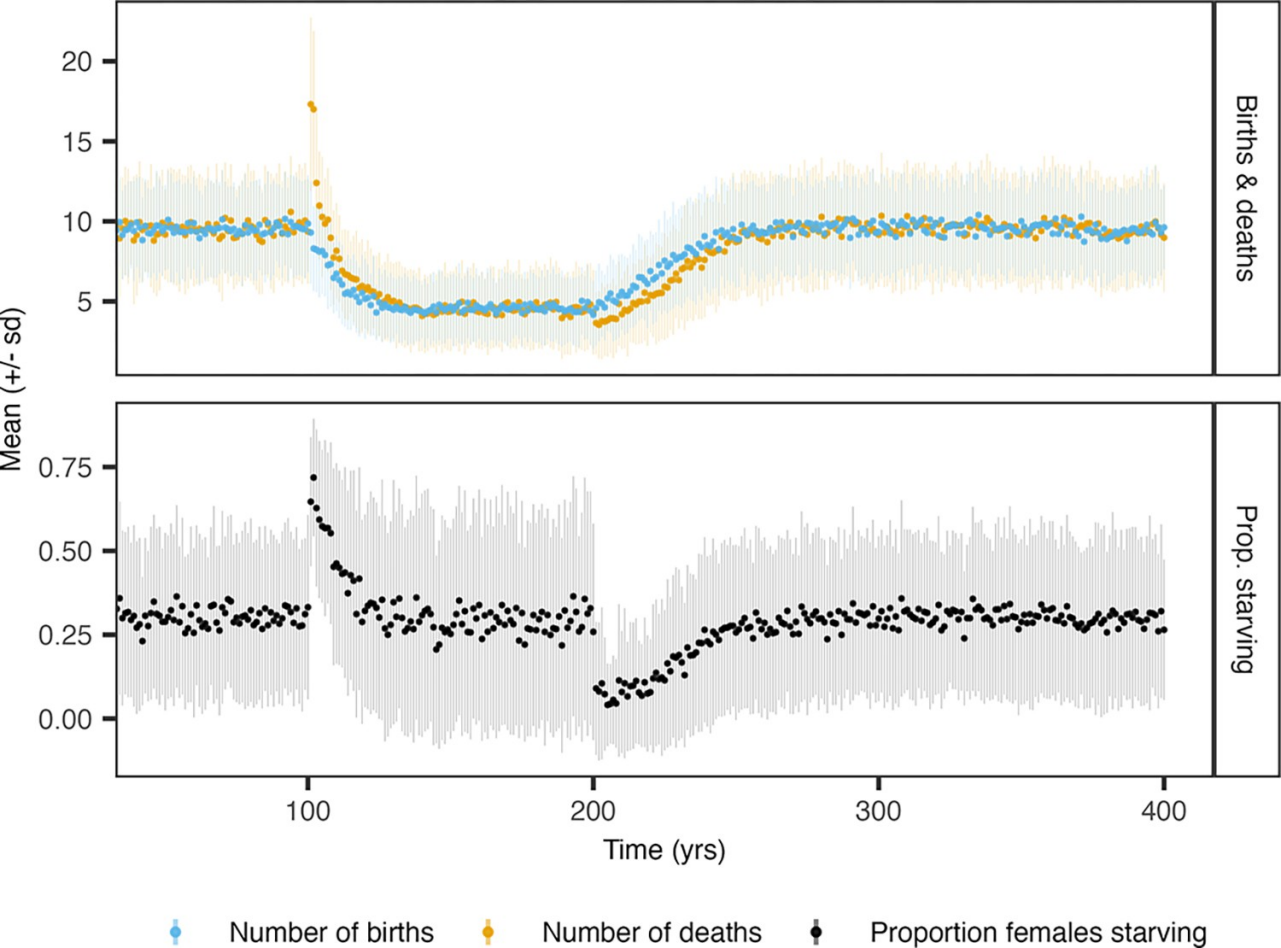
Total number of births and deaths per year (top panel) and yearly proportion of females that died from starvation (bottom panel) for the simulated *Zc* population at SOAR, in response to the onset of MFAS at *t* = 100 y. Disturbance by MFAS led to cessation of foraging. At *t* = 200 y., MFAS stopped and the population recovered. The associated trajectories of population abundance are shown in left panels of Fig 3. Females are categorized as starving if, upon death, their body condition was below the starvation body condition threshold. Points and line ranges represent means and standard deviations from 100 replicate simulations, respectively.

After the onset of MFAS, there was an immediate peak in the yearly number of *Zc* deaths from all causes and in the proportion of *Zc* females that died from starvation (Fig 4). In the first years after the onset of MFAS, when the population declined most strongly (Fig 3), the number of *Zc* calves born was only slightly lower than before MFAS started (Fig 4). In the stationary population under disturbance, the number of births and deaths were approximately half of those in the undisturbed population, reflecting the 50% reduction in population size (Fig 3, top left panel). Approximately 30% of *Zc* females died from starvation in a stationary population, both with and without disturbance from MFAS. Upon cessation of MFAS, there was an immediate reduction in the proportion of starving females and the number of deaths per year, which led to population recovery.

**Fig 5:**
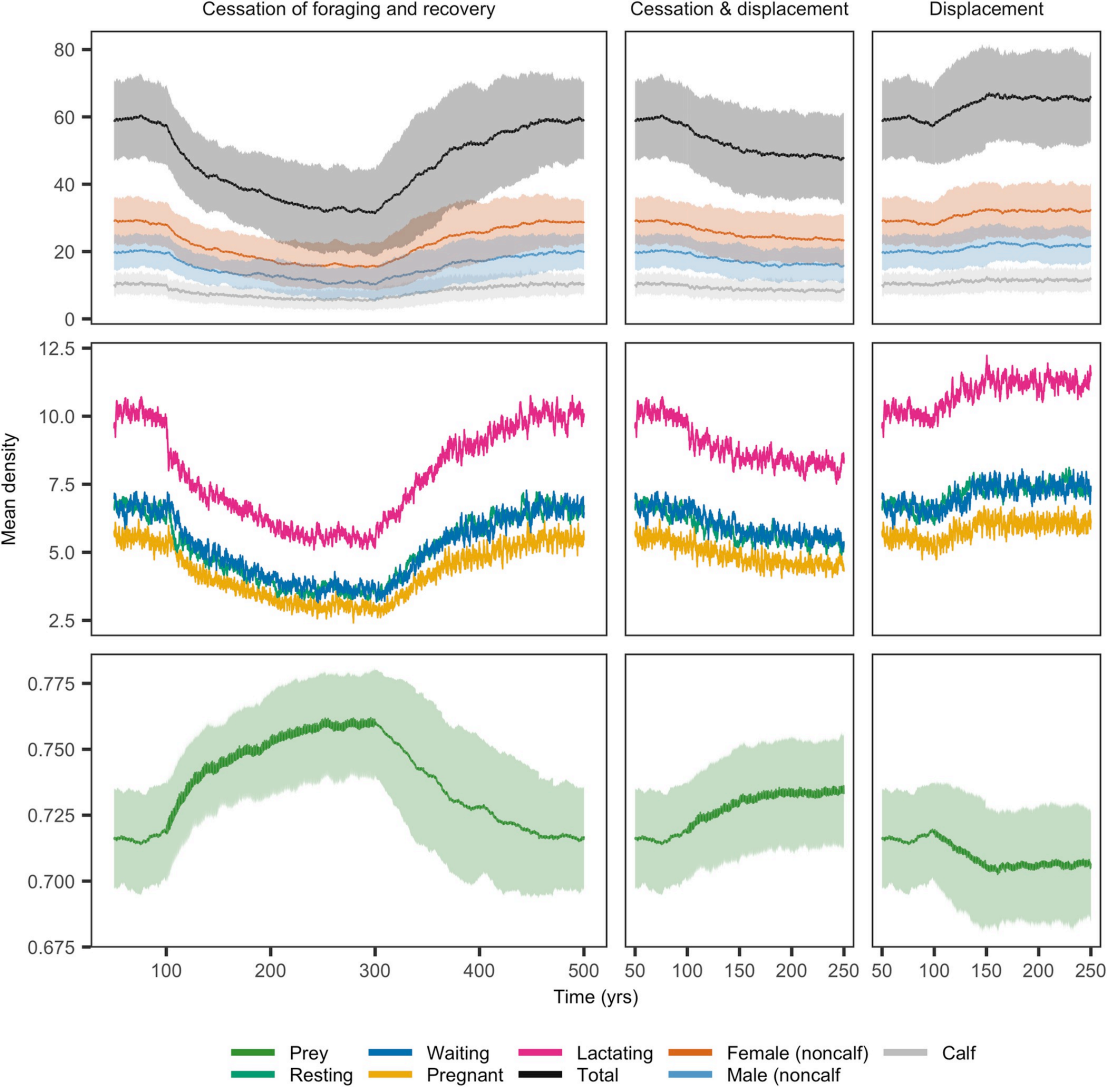
Population dynamics of *Md* at AUTEC in response to onset of disturbance at *t* = 100 y. Disturbance either led to cessation of foraging (left panels), displacement from range (right panels) or both (center panels). In the first scenario, the recovery of the population after cessation of MFAS disturbance at *t* = 300 y is plotted. Lines and shaded areas indicate mean and standard deviation of 100 replicate simulations, respectively. Middle rows show reproductive classes of females and standard deviations are omitted here for sake of presentation. Note the differences in time scales between panels. All parameters at default values (Table S3 in S1 File).

For *Md* at AUTEC, the effect of disturbance from MFAS on mean population abundance was largest for the cessation-of-foraging behavioral response, with an approximate 45% reduction of mean population size (Fig 5). MFAS-induced displacement led to a slight increase of mean *Md* population abundance (from 59 to 65 individuals). The displacement and cessation-of-foraging response induced an effect on mean population abundance that was intermediate between the effect of the two responses in isolation. The decline of mean *Md* population abundance was much slower than that of *Zc* at SOAR: half of the initial decrease occurred within around 24 years, on average, and it took another 123 years before the population was within 1 per cent of the mean stationary population abundance under disturbance (Fig 5). The onset of MFAS at AUTEC also changed prey density and its variation, but the size of this effect was much smaller than at SOAR. The recovery of the *Md* population after MFAS ceased was slower than for *Zc* and took approximately 156 years. The first half of the population recovery took around 59 years, corresponding to an annual growth rate of 0.59%.

The sensitivity of model outcomes to movement patterns was investigated by adjusting the elements of the transition matrix (**Q**) and changing the attack rate ratio ${\phi_{R}}_{high}$. The **Q**-matrix was adjusted so that a larger fraction of the population was present on the range under baseline conditions with no MFAS (Table 1). For *Zc* this adjustment meant that 85% of the population was on-range (70% in the western range areas and 15% in the eastern areas). For *Md*, 81% of the population was on-range, with 66% and 15% in the western and eastern range areas.

**Fig 6:**
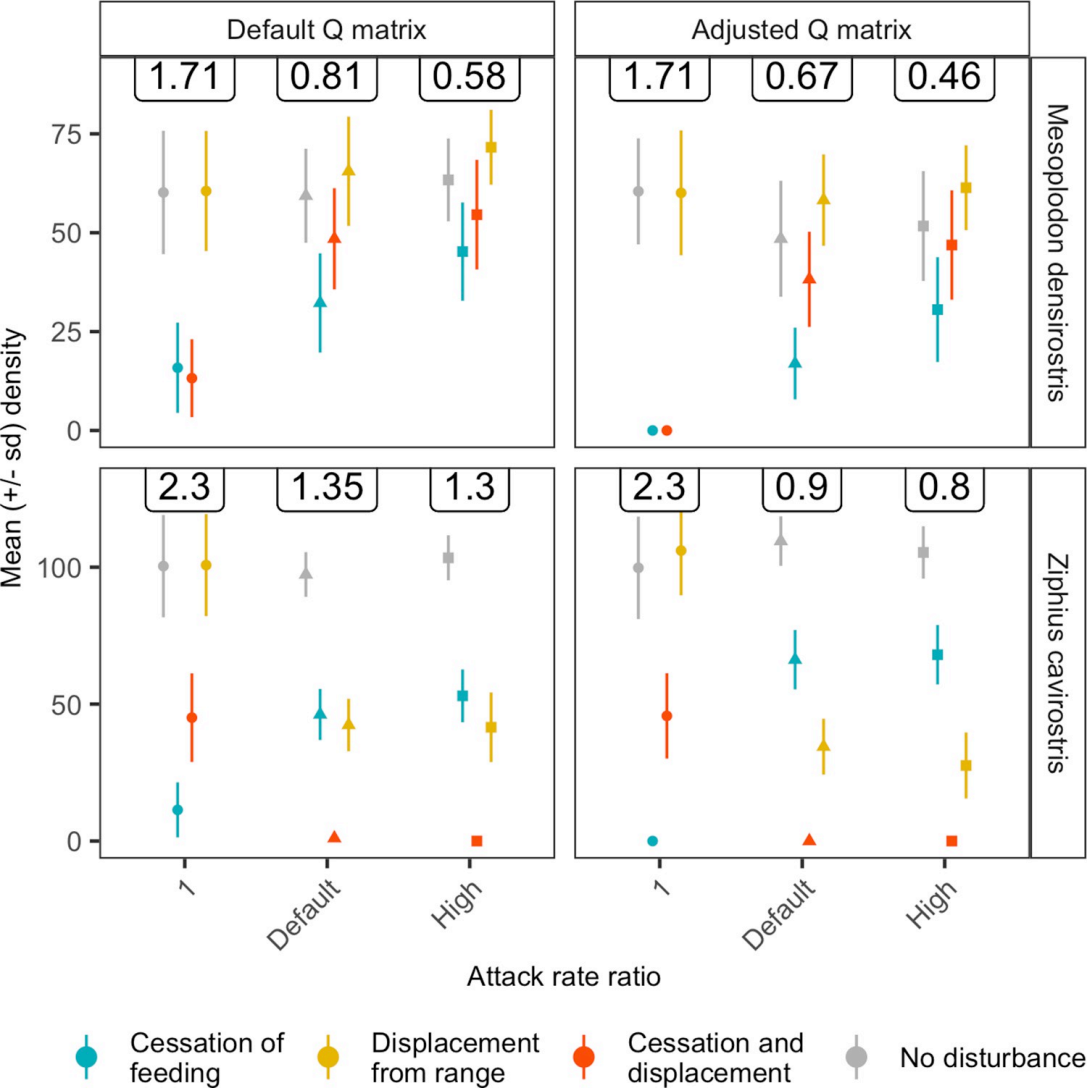
The effect of disturbance, differences in habitat quality (quantified by the attack rate ratio $\phi_{R_{high}}$) and movement patterns (Q–matrix) on population abundance. The maximum prey density in absence of whale foraging (*R*_*max*_) was adjusted for each combination of attack rate ratio and **Q**–matrix, to obtain an approximately equal undisturbed population abundance. Labels indicate the value of *R*_*max*_ used. Points show time-averaged values of mean population abundance across different replicate simulations. Line ranges show time-averaged values of the standard deviation of population abundance across different replicate simulations. Attack rate ratio ($\phi_{R_{high}}$) for *Zc* and *Md* equaled 13.3 and 5.0 by default, and 20 and 10 under the ‘high’ attack rate ratio scenario. All other parameters at default values (Table S3 in S1 File).

For both species, the relative pattern of population abundance across the different disturbance scenarios was robust to changes in **Q** and ${\phi_{R}}_{high}$ (Fig 6). For *Md*, MFAS-induced cessation of foraging had the largest negative effect on population abundance, while displacement by MFAS led to a slight increase in population abundance. However, for ${\phi_{R}}_{high}=1.0$ (i.e., no area-specific differences in habitat quality), displacement did not affect *Md* abundance and the other two scenarios had a similar effect. For ${\phi_{R}}_{high}=1.0$, the adjusted **Q**-matrix led to extinction of the *Md* population if the response to MFAS disturbance included cessation of foraging. For *Zc* and ${\phi_{R}}_{high}>1.0$, displacement from SOAR had a larger impact on population abundance than cessation of foraging, and this pattern was especially pronounced for the adjusted **Q-**matrix. The combination of displacement and cessation of foraging led to extinction of the *Zc* population. For ${\phi_{R}}_{high}=1.0$, MFAS-induced displacement did not affect the *Zc* population abundance and reduced the effect of cessation of foraging, which on its own had a larger effect on population abundance than for ${\phi_{R}}_{high}>1.0$.

**Fig 7:**
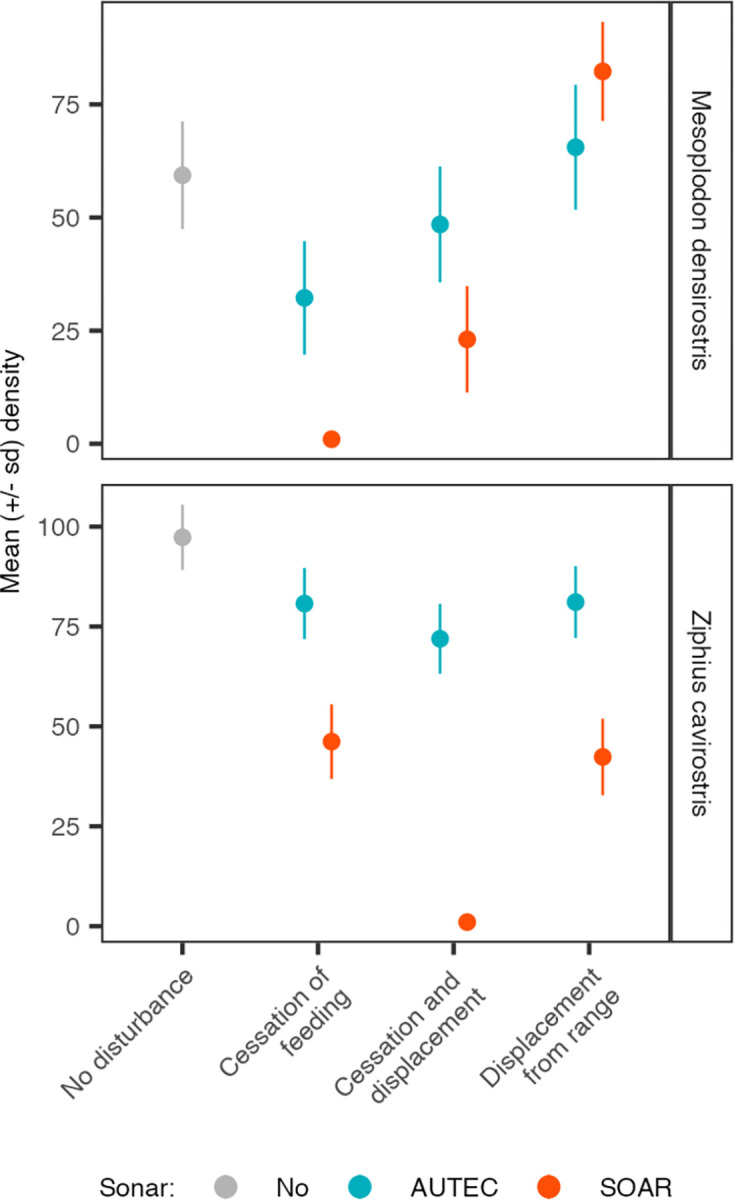
The interaction between species, the pattern of MFAS use and the response to MFAS. By default, the time series of MFAS from AUTEC was applied to the population of *Md* (blue symbols top panel) and the MFAS signal from SOAR was applied to *Zc* (red symbols bottom panel). This plot shows the effect of the hypothetical scenario in which *Md* would be exposed to the MFAS pattern at SOAR (red symbols; top panel) and *Zc* to MFAS at AUTEC (blue symbols; bottom panel). For both species, MFAS as used at SOAR had a stronger effect on population abundance compared to MFAS as used at AUTEC. All other parameters at default values (Table S3 in S1 File).

For both species, applying the pattern of MFAS use from SOAR had the largest effect on population abundance (Fig 7). This pattern led to the extinction of *Md* in the cessation-of-foraging scenario, and to an even higher increase in population abundance in the displacement scenario. Conversely, applying the MFAS time-series from AUTEC to the *Zc* population decreased the disturbance effect on population abundance and ensured persistence of *Zc* under all behavioral scenarios considered.

**Fig 8:**
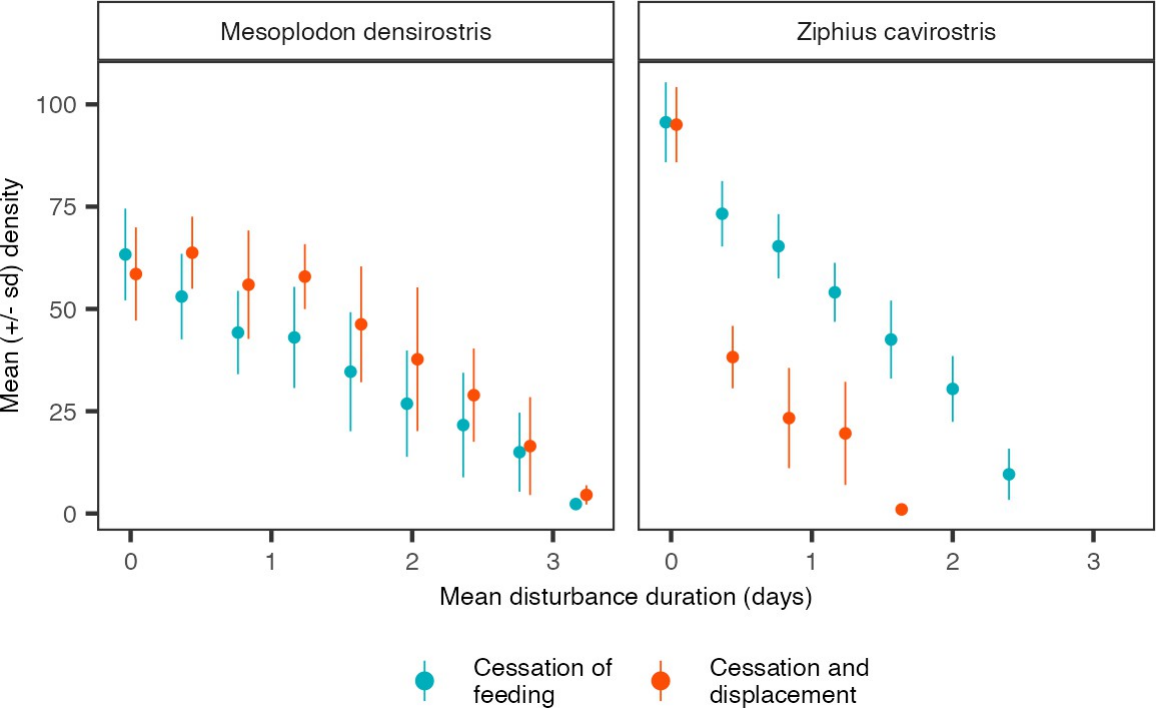
The effect of increasing the mean duration of cessation of foraging on population density of *Zc* and *Md* for the two relevant disturbance scenarios. Population density at zero mean disturbance duration represents undisturbed population density. Upon disturbance, duration of cessation of foraging was randomly determined from an Erlang distribution with shape parameter 2 and scale parameter equal to half the mean disturbance duration. By default, a mean disturbance duration of 1.5 days was used, corresponding to a scale parameter of 0.75. Points represent time-averaged values of mean population abundance across replicate simulations. Lines represent time-averaged values of the standard deviation of population abundance across replicate simulations. Because population abundances were averaged over time, only eight replicate simulations were used. All other parameters at default values (Table S3 in S1 File).

Increasing the mean duration of cessation of foraging led to a further decrease in population abundance of both species but did not change the relative pattern of the effect of MFAS disturbance (Fig 8). For *Zc*, cessation of foraging and displacement led to lower population abundances than cessation of foraging alone, irrespective of the mean disturbance duration. For *Md*, displacement by MFAS reduced the effect of cessation of foraging across all disturbance durations considered.

### Disturbance effects on life history and body condition

For both species, life history statistics relating to the onset of reproduction were not affected by disturbance from MFAS and were similar across all behavioral response scenarios (Fig S7 in S1 File). Note that these results relate to females from stationary populations subject to a given disturbance regime, and not to the transient phase of population decline immediately after the onset of MFAS. Regarding the onset of reproduction, we distinguished between female age at first receptive, female age at first reproduction and female age at weaning first calf. Because these data represent females from stationary populations, mean lifetime reproductive output was equal to 2 (counting male and female calves born to each female; Fig S8 and Table S6 in S1 File). The mean number of calves weaned per female was slightly higher for *Zc* compared to *Md*, indicating that calf survival was slightly lower in the latter species.

Disturbance from MFAS did not affect body condition of females in the stationary populations (Fig S9 and Table S6 in S1 File) subject to a given disturbance regime. For both species, body condition was lowest for lactating females and highest for calves, although there was large variation in this latter class. For *Zc*, resting females had lower body condition than pregnant and waiting females.

## Discussion

The effects of disturbance from MFAS on population density were driven by the interaction between the intensity of MFAS use, the relative habitat quality of the different areas and the behavioral response to MFAS. MFAS use was more frequent on SOAR, and this pattern had a greater effect on both populations than the pattern of sonar use at AUTEC (Fig 6). However, whether MFAS led to an increase or a decrease in population abundance was determined by the behavioral response to MFAS in combination with modelled differences in habitat quality between areas. The majority (81%) of *Md* individuals exposed to MFAS resided on the western part of AUTEC, which was an area with high habitat quality. Individuals displaced from western AUTEC moved to the area north west of the range, also a high-quality area. Therefore, for most modelled *Md* individuals, displacement by MFAS did not impact their energy intake. In fact, displacement likely increased the fraction of the *Md* population residing in high-quality areas, because without displacement, some modelled individuals would have moved to the lower quality area south of the range. This explains the slight increase in *Md* population abundance that was observed when MFAS led to displacement only and habitat quality differed between areas.

In contrast, *Zc* individuals that were displaced from SOAR by MFAS always moved to an area of lower habitat quality, in which they resided for an average of 7.2 days (1/0.1397; Table 1). Assuming that there are differences in habitat quality between areas, displacement had a similar effect on population abundance to MFAS-induced cessation of foraging with a default average duration of 1.5 days. The combined effect of both behavioral responses was additive and resulted in the extinction of the modelled *Zc* population. Likely, the decline of the *Zc* population in response to displacement by MFAS will be lessened if *Zc* whales were displaced to a high-quality area.

**Fig 9:**
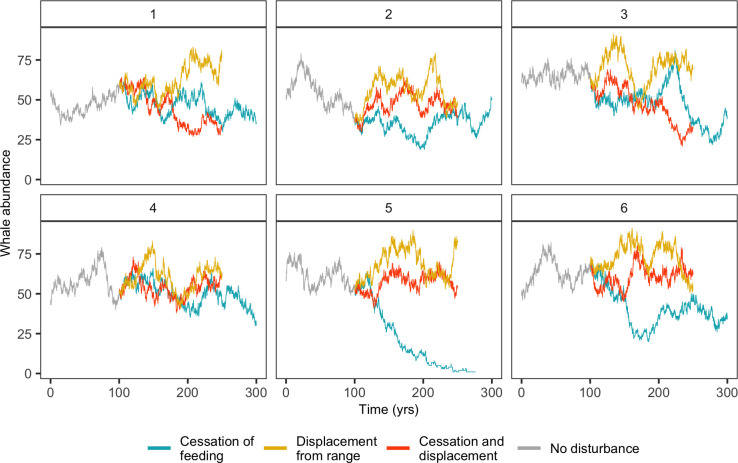
Six replicate simulations (panels) of the modelled population abundance of *Md* in response to MFAS disturbance for three different behavioral response scenarios (colors). These simulations contribute to the mean population abundance of *Md* as shown in Fig 5. As opposed to mean population abundance, output from individual simulations give a better idea of the variation of population abundance across time and between replicate simulations. All parameters at default values (Table S3 in S1 File).

The modelled population trajectories show substantial fluctuations around a carrying-capacity level that are a consequence of the stochastic processes in the model, these include mortality (life expectancy was randomly determined), onset of pregnancy, sex determination of offspring and movement. The large variability in population abundance has important implications for the ability to detect population-level effects of MFAS-induced behavioral disruptions. In our modelled population, the effects of disturbance were studied using 100 replicate simulations, which resulted in an unequivocal change in population abundance (Figs 3 and 5). However, the MFAS-induced changes in population abundance are much harder to detect in the population trajectory of a single replicate simulation (Fig 9), where there may be episodic reversals of the predicted effect of disturbance, and population abundance may temporally increase when the population is exposed to MFAS. This was especially true for *Md*, where population abundance was lower and the predicted effect of MFAS was smaller. In addition, the estimated low rate of population growth implies that frequent monitoring on a decadal scale would be required to detect any population change [68].

The ability to detect population-level effects will likely be further reduced if the effects of environmental variability or seasonality [69–71], were introduced into the model. For example, productivity in the bathypelagic zone varies considerably in time and space, and is strongly influenced by shifting deep-water currents that bring in nutrient-rich waters [53, 60, 72]. Incorporating these sources of variability into the model will further increase the predicted variation in population density, and may thereby mask effects of disturbance on population abundance.

We did not find any effect of disturbance on individual life-history characteristics once the simulated populations had attained a stationary state. However, during the initial decline phase following the onset of sonar, females had reduced reproductive output and experienced increased mortality due to starvation (Fig 4). The increase in starvation mortality following the onset of MFAS indicates a decline in body condition, a result that was also found by [51]. Monitoring programs that focus on individual vital rates would therefore need to start well in advance of any planned disturbing activities to ensure that sufficient baseline observations to detect any changes are available [73, 74].

Although we used realistic patterns of MFAS disturbance derived from passive acoustic data, there are limitations and uncertainties within the direct measurements of movement rates and prey availability, as well as several remaining sources of uncertainty that are not explicitly accounted for in the model. For example, the differences in habitat quality we used were based on empirical measurements of prey availability that had limited spatial and temporal coverage [53, 60, 72] compared to the entire habitat used by these whales. At SOAR the assumption of prey quality declining so dramatically in off-range areas is based on prey sampling in a limited area on a single day [60], and is likely not reflective of the much larger area that encompasses the off-range area where whales can, and often do, move [30, 49]. The same holds for the tracking data used to estimate the transition rates of the movement model. These were derived from a limited number of whales over time span of up to 89 days and are potentially biased because the tagged animals were exposed to sonar during their transmission duration [30, 31, 42]. In addition, the derived transition rates do not necessarily reflect the area-specific differences in habitat quality, as the distribution of animals across the different areas bears no relation to the area-specific habitat quality values. Unaccounted uncertainty also exists with regard to energetic aspects of the model, because a number of parameters could not be estimated directly for beaked whales (S1 File). In particular, there is uncertainty regarding the ability of beaked whales to withstand periods of food deprivation, because beaked whale blubber tissue is mainly composed of wax esters that are relatively inert and probably not easily mobilized compared to the triacylglycerols found in the blubber tissue of most other cetacean species [75–77]. Our model suggests that movement patterns and area-specific differences in habitat quality drive the modelled population response to disturbance, targeted research efforts are therefore required to refine these model inputs and obtain more accurate predictions of the aggregate effects of MFAS-induced behavioral disturbance on beaked whale populations [78].

Another key uncertainty is the variation in MFAS exposure over time and between individual whales. The standardized sonar area implicitly accounts for sonar intensity in that louder sonar should be detected over a larger area. However, it does not explicitly account for sonar source level, distance to sonar source, number of sonar sources, sonar source type, or other naval training activities, all of which may influence behavioral response [43, 79]. Similarly, we assumed that off-range areas were free of sonar, although in reality sonar can be detected throughout the wider area [30] and this will likely vary between AUTEC and SOAR, due to environmental and topographical differences between the two locations. In addition, we have assumed that all modelled individuals were regularly exposed to MFAS, as the same movement rates were applied to all whales. This assumption is supported by the observation that beaked whales form localized populations with limited ranges and low dispersal rates [35, 41, 80, 81]. However, there may be other intra-individual differences in exposure to MFAS, in addition to the random variation inherent in the Markovian movement model. If some individuals only rarely visit Navy training ranges or only during certain times of the year, our model will only capture the behavior of a subset of a larger (meta)population and therefore represent a worst-case scenario. For example, a similar integrative modelling study of eastern North Pacific blue whales (*Balaenoptera musculus*) by Pirotta et al. [78] showed there was no effect of MFAS disturbance on vital rates (reproduction and survival), due to the limited overlap in space and time between whales and military activity. A good understanding of the full geographical range of the population that is being exposed to disturbance, as well as intra-individual variation in dive behavior and exposure history, is essential for improving predictions about the population effects of disturbance [49, 82, 83].

Density-dependent population models have revealed that nonlethal disturbance effectively decreases population carrying capacity and that compensatory effects on individual life history can occur through a disturbance-induced release of top-down control of prey [this study, 51]. This is a result of the increased prey density that is a consequence of the relaxation in predation pressure. For simplicity, we have assumed that prey dynamics are entirely determined by beaked whale predation. However, in reality, there are many other predators on the deep-water squid that are the principal prey of beaked whales. If the dynamics of any of these predators are similar to that of *Md* and *Zc*, they may become more abundant as a result of the increase in prey density and thus reduce the ‘surplus’ production of prey that is available to the two beaked whale species.

Hin et al. [51] used the same energy budget model as this study, but parameterized for long-finned pilot whales to show that nonlethal disturbances that resulted in cessation of foraging for multiple, consecutive days each year led to increased mortality among young females that were nursing their first calf and increased reproductive output of older females. In addition, nonlethal disturbance increased mean body condition in the population and led to earlier first reproduction. The fact that we did not find any effect of disturbance on individual life history and female body condition probably relates to the specific disturbance scenarios used here, which involved multiple disturbance events of short duration throughout the year that only affected a subset of the population. In contrast, Hin et al. [51] purposely used extreme disturbance scenarios, in which the entire population was exposed to long-lasting disturbance that continued for up to 40 consecutive days each year.

Our study indicates that the magnitude of the population effect of nonlethal disturbance likely depends on a multitude of factors, such as the spatial distribution of prey resources, the type and severity of the behavioral response, baseline movement patterns and the intensity and temporal pattern of the stressor (MFAS use in our case study). This underscores the importance of a multidisciplinary approach that integrates results from multiple lines of research into a single assessment framework [78]. In particular, our study highlights that a good understanding of the dynamics and spatio-temporal variation in prey resources is critical, as this will determine the ability of individuals to compensate for the energetic losses due to disturbance [83, 84]. Such knowledge will become increasingly important as climate-induced changes in species’ habitats accelerate [78, 85, 86].

## Supporting information

S1 File Supplemental material. (PDF)
