# Supplementary material for: Using individual-based bioenergetic models to predict the aggregate effects of disturbance on populations: A case study with beaked whales and Navy sonar
Source: PLoS One. 2023 Aug 31;18(8):e0290819. doi: 10.1371/journal.pone.0290819 (PMC10470956; doi:10.1371/journal.pone.0290819)
Supplement: S1 File — (PDF) [file pone.0290819.s001.pdf]

Supplemental material

Using individual-based bioenergetic models to predict the aggregate effects of disturbance on populations: a case study with beaked whales and Navy sonar

Vincent Hin, André M. de Roos, Kelly J. Benoit-Bird, Diane E. Claridge, Nancy DiMarzio, John W. Durban, Erin A. Falcone, Eiren K. Jacobson, Charlotte M. Jones-Todd, Enrico Pirotta, Gregory S. Schorr, Len Thomas, Stephanie Watwood, John Harwood

1. Energy budget model

We used the energy budget model originally developed by De Roos et al., (2009) for ungulates and adapted for the long-finned pilot whale (*Globicephala melas*) by Hin et al. (2021, 2019). This model describes individual energy acquisition from prey feeding (and milk suckling for calves) and utilization of energy on metabolism, growth in body size, pregnancy and lactation, as a function of an individual's state. All individual state variables are listed in Table S1 and model equations are given in Table S2.

Table S1: Individual state variables

State variable	Symbol	Equation	Unit
Age	a	-	day
Time since conception	τ_p	-	day
Structural length	l	$l_\infty - (l_\infty - l_b)e^{-ka}$	cm
Structural length fetus	l_p	$l_b \frac{\tau_p}{T_P}$ for $0 \leq \tau_p \leq T_P$	cm
Structural mass	S	$\omega_1 l^{\omega_2}$	kg
Reserve mass	F	-	kg
Total mass	W	$\begin{cases} S + F + S\left(l_p(\tau_p)\right) & \text{pregnant} \\ S + F & \text{otherwise} \end{cases}$	kg
Maintenance mass	W_M	$\begin{cases} S + \theta_F F + S\left(l_p(\tau_p)\right) & \text{pregnant} \\ S + \theta_F F & \text{otherwise} \end{cases}$	kg
Body condition	-	F/W	-

Individual whales are composed of structural mass and reserve mass. Structure represents tissue that requires maintenance and cannot be used as energy store (e.g. bones and organs), while reserves can and require little or no maintenance (Kooijman, 2009). Structural length of free-living individuals is a fixed Von Bertalanffy function of age (Table S1) with parameters length at birth (l_b), asymptotic length (l_∞) and Von Bertalanffy growth constant k. We do not consider sexual dimorphism in structural size for the two beaked whale species. Growth in structural size is non-plastic: growth rate and asymptotic size do not vary with rate of energy assimilation. Therefore, this energy budget model describes demand-driven growth, which was assumed more appropriate for mammals than the supply-driven Dynamics Energy

Budget (DEB) formulation of Kooijman (2009). Demand-driven growth implies that rates of energy expenditure are independent of the energy acquired from feeding, because growth rate does not depend on energy intake. Instead, individuals regulate their body condition (fraction of reserves to total mass) by adjusting their feeding effort (see below). For fetuses, structural length is a linear function of time since conception (τ_p). Structural mass is directly related to structural length through a length-weight relationship and is therefore a fixed function of age too. Total body mass equals the sum of structural mass and reserve mass and includes structural mass of any fetus. Maintenance body mass is a weighted measure of body mass that discounts the mass-specific contribution of reserve mass to metabolic rate through parameter θ_F (Table S1).

Energy acquisition from prey feeding (I_R) and milk suckling (I_L) are directly used to cover all energetic costs. All individuals spend energy on maintenance costs (C_M) and growth (C_G). Gestation (C_p) and lactation (C_L) costs are paid by, respectively, pregnant and lactating females only. Females can be simultaneously pregnant and lactating. If energy acquisition is insufficient to cover all energy expenditures, additional energy is made available by catabolizing reserves. Vice versa, if energy intake exceeds energy expenditure, the surplus energy is stored as reserves (anabolism). Consequently, dynamics of reserve mass (d*F*/d*a*) depend on the rates of energy intake and expenditure, which in turn depend on the reproductive status of an individual:

$$\frac{dF}{da} = \begin{cases} \varepsilon_i^{-1}(I_R + I_L - C_G - C_M) & \text{Calves} \\ \varepsilon_i^{-1}(I_R - C_G - C_M - C_P) & \text{Pregnant female} \\ \varepsilon_i^{-1}(I_R - C_G - C_M - C_L) & \text{Lactating female} \\ \varepsilon_i^{-1}(I_R - C_G - C_M - C_P - C_L) & \text{Pregnant and lactating female} \\ \varepsilon_i^{-1}(I_R - C_G - C_M) & \text{Other} \end{cases} \qquad (1)$$

With the reserve mass at birth as initial condition $F(0) = F_b$. In eq. (1), the anabolic $\varepsilon_i = \varepsilon_+$ if $\frac{dF}{da} > 0$ and catabolic $\varepsilon_i = \varepsilon_-$ if $\frac{dF}{da} < 0$ conversion parameters account for the efficiency of storing and utilizing reserves.

The function describing energy assimilation rate from prey feeding (I_R in MJ day^{-1}) consists of three different components. The first component is a type II functional response of prey density *R*, with a maximum consumption rate that depends on structural surface-area $S^{2/3}$ and the handling time parameter *h*. The attack rate parameter of the functional response (ϕ_R^j) varies between areas (*j*) (Table S2). The second component represents the feeding effort ($\frac{1}{1+e^{-\eta(\rho W/F-1)}}$) and describes the fraction of time spent foraging. The feeding effort is modelled as a decreasing sigmoidal function of body condition (F/W) that equals 0.5 at the target body condition $\rho = 0.3$. The last component of prey foraging models the prey feeding efficiency (capture success), which is lower for younger whales and gradually increases with age according to a hyperbolic function ($\frac{a^\gamma}{T_R^\gamma + a^\gamma}$).

The lactation rate is proportional to calf structural surface-area ($\phi_L\, S^{2/3}$) and subject to the decrease in feeding effort of the calf in response to calf body condition. The lactation rate is

modified by a hyperbolic function of maternal body condition (F_m / W_m) that equals zero if maternal body condition falls below ρ_s and one at $F_m/W_m = \rho$. Furthermore, beyond age T_N, milk demand by the calf declines as the calf approaches weaning age (T_L).

Costs for structural growth (C_G) are obtained from the derivative of structural mass with respect to age multiplied by costs of growth parameter σ_G. Metabolic costs follow a quarter power relationship of maintenance body mass (W_M) following Kleiber (1975) and Lockyer (2007). Pregnancy costs (C_P) are proportional to fetal growth in structural mass. Fetal survival is assumed to be 0.8 over the whole of the gestation period and probability of abortion is independent of female state. Lactation costs (C_L) for the female are proportional to the lactation rate (I_L) of the calf, accounting for the efficiency of milk production and consumption through parameter (σ_L).

Table S2: Equations of the energy budget model for an individual whale

Function	Equation	Description
$I_R(a,R,S,F,W)$	$\frac{\phi_R^j R\, S^{2/3}}{1+\phi_R^j\, h\, R}\cdot\frac{1}{1+e^{-\eta(\rho W/F-1)}}\cdot\frac{a^\gamma}{T_R^\gamma+a^\gamma}$	Energy assimilation rate from prey feeding in area j
$I_L(a,S,F,W,F_m,W_m)$	$\phi_L S^{2/3}\left[\frac{(1-\xi_m)(F_m-\rho_s W_m)}{(\rho-\rho_s)W_m-\xi_m(F_m-\rho_s W_m)}\right]_+ \cdot \frac{1}{1+e^{-\eta(\rho W/F-1)}}\min\left(1,\left[\frac{1-\frac{a-T_N}{T_L-T_N}}{1-\xi_c\frac{a-T_N}{T_L-T_N}}\right]_+\right)$	Energy assimilation rate from milk
$C_M(W_M)$	$\sigma_M W_M^{3/4}$	Field metabolic costs
$C_G(S)$	$\sigma_G\omega_1 k(l_\infty-l)\omega_2 l^{\omega_2-1}$	Somatic growth costs
$C_P(\tau_p)$	$\sigma_G\omega_1\omega_2\left(\frac{l_b}{T_P}\right)^{\omega_2}\tau_p{}^{\omega_2-1}$, for $0\le\tau_p\le T_P$	Pregnancy costs
$C_L(F,W,a_c,S_c,F_c,W_c)$	$I_L(a_c,S_c,F_c,W_c,F,W)/\sigma_L$	Lactation costs
$D_b(a)$	$\alpha_1 e^{-\beta_1 a}+\alpha_2 e^{\beta_2 a}$	Mortality rate calves and weaned females
D_b	μ_{male}	Mortality rate weaned males
$D_s(F,W)$	$\mu_s\left(\frac{\rho_s W}{F}-1\right)$, if $F<\rho_s W$	Starvation mortality rate
F_{preg}	$\rho_s W+F_{neo}$	Pregnancy threshold
F_b	$\frac{\rho_s\omega_1 l_b{}^{\omega_2}}{1-\rho_s}$	Reserve mass at birth

Mortality and survival

Calves and weaned females experience age-dependent mortality rate (*$D_b(a)$*) according to a Siler model with three components: i) constant background mortality, ii) early life mortality that declines with age and iii) senescence mortality that increases with age (Barlow and Boveng, 1991; Siler, 1979). Weaned males experience constant background mortality only, with rate μ_{male}. In addition, individuals experience additional starvation-induced mortality if their body condition falls below the starvation body condition threshold $\rho_s = 0.15$. The starvation mortality rate is a hyperbolic function that increases with decreasing body condition (Table S2). The survival function *H(a)* is the integrated outcome of both starvation and (age-dependent / background) mortality: $H(a) = e^{-\int_0^a \left(D_b(\alpha)+D_s\left(F(\alpha),W(\alpha)\right)\right)d\alpha}$, which is a decreasing function of age with *H(0)* = 1.0. In natural populations, there is variation in age at death even in the absence of starvation mortality. To capture this variation, each individual was assigned a survival threshold: a uniform random number between 0 and 1. If *H(a)* falls below this threshold the individual was considered death and removed from the population.

Reproduction

Whales are assumed to initiate pregnancy when the absolute amount of reserves exceeds the ‘pregnancy threshold’, defined as $F_{preg} = \rho_s W + F_{neo}$, where $\rho_s W$ is the reserve mass threshold below which starvation mortality occurs. Once the female becomes receptive she is assigned the 'waiting' status, as she awaits the onset of pregnancy. Each time a female enters the waiting period, its duration is randomly determined from an exponential distribution with a mean of 445 days. Gestation last T_G days and during gestation fetuses experience a daily mortality rate of μ_F, which is independent of the body condition of their mother. Hence, termination of pregnancy by the female was not considered. Fetal growth in structural length is a linear function of time since conception (Table S1). Fetuses do not contain any reserve mass, as they are directly linked to the reserves of their mother. Upon parturition, the minimum amount of reserves to offset starvation mortality (F_b) is transferred from the mother to the neonate (Table S2). Lactation lasts T_L days and females can reinitiate the next pregnancy (enter the waiting period) during the last T_G days of the lactation period if their reserve mass exceeds F_{preg}. This restriction is to prevent females from having two calves simultaneously. Waiting females can become pregnant during the last phase of lactation if the waiting period happens to be shorter than the gestation period (T_G).

Prey dynamics

Prey dynamics (d*R*/d*t*) are modelled using a semi-chemostat approach, with a fixed prey productivity term δR_{max} and a first-order prey removal term δR. In absence of predation by whales, prey growth rate is a linear decreasing function of prey density and prey density equilibrates at $R = R_{max}$. During analysis, the maximum prey density R_{max} was varied to obtain a beaked whale population abundance that matched observed densities (around 100 *Zc* and 60 Md individuals). Total consumption of prey by all whales decreases prey biomass. For sake of simplicity, prey biomass (MJ m^{-3}) is expressed in MJ assimilated energy and therefore includes any overheads and efficiency of prey assimilation by whales. The volume scalar *V* converts the

volume unit of prey density (m^3) to the volume unit of whale density (V m^3). The ordinary differential equation of prey biomass equals:

$$\frac{\mathrm{d}R}{\mathrm{d}t} = \delta(R_{max} - R) - V^{-1}\sum_{i}^{N} I_R(a_i, R, S_i, F_i, W_i) \quad \text{eq (2)}$$

2. Parameterization

Little is known about the biology of beaked whales compared to other odontocetes and several parameter values for *Globicephala melas* (hereafter *Gm*) from Hin et al. (2021, 2019) are therefore retained for *Zc* and *Md*. Model parameters and their sources are summarized in Table S3. Derivation of several parameters is described below.

Asymptotic length

Length estimates for beaked whales are given in Mead (1984) and, more recently, MacLeod (2006). According to Mead (1984), the maximum length of females *Zc* is 754 cm. However, Heyning (1989) notes that reported lengths > 7 m for *Zc* probably represent misidentified individuals, as these are all derived from individuals that co-occurred with larger beaked whale species (*Berardius* spp. and *Hyperoodon*). Indeed, the vast majority (93%) of *Zc* individuals reported in MacLeod (2006) are under 7 m in length and according to Heyning (1989), the largest accurately identified *Zc* measured 693 cm in length. Data from catches of *Zc* in the North Pacific indicate a maximum length for females of 670 cm (MacLeod, 2006; Nishiwaki and Oguro, 1972). Because there is no significant different in total length between sexes of *Zc*, we adopt 670 cm as our asymptotic length estimate for *Zc* (Table S3). For *Md* we adopt an asymptotic length of 475 cm, based on the observation by MacLeod (2006) that *Md* rarely reaches over 4.8 m in length.

Size at birth

For *Zc*, we adopt the mean length at birth estimate of Mead (1984), which is 270 cm. Following (New et al., 2013), length at birth for *Md* was set to 198 cm. This value also falls in between the length of longest fetus (190 cm) and length of shortest calf (261 cm), as reported by Mead (1984).

Von Bertalanffy growth constant

The Von Bertalanffy growth constant was calculated by rewriting the growth equation and using estimates of mean age (a_m) and length at sexual maturity (l_m).

$$k = \frac{-1}{a_m} \ln\left(\frac{l_\infty - l_m}{l_\infty - l_b}\right)$$

For *Zc*, Mead (1984) reports mean length at sexual maturity of 580 cm and age at sexual maturity was estimated at 2250 days, which give k = 0.000663 for Zc. Applying the same procedure for *Md* with mean length and age at sexual maturity of, respectively, 378 cm and 3285 days (9 yrs; Claridge, 2013) gives k = 0.000319.

Structural length – mass relationship

To estimate the scaling constant and exponent of the length – mass relationship of structure, we collect length-weight data from stranded *Zc* and *Md* individuals. Data from emaciated or starved individuals were excluded from analysis, as well as those reported to be in a moderate or

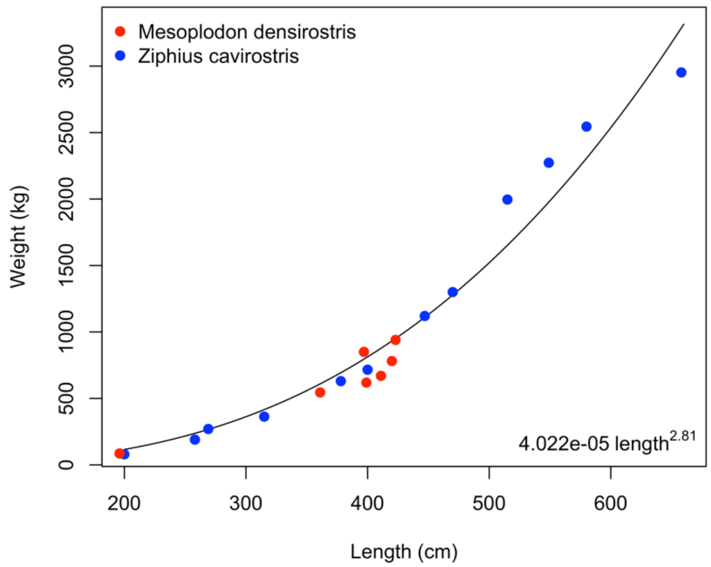

Figure S1: Length-weight relationship of various stranded beaked whales

advanced state of decomposition. Because of the limited number of data points, we pooled observations from both species and fitted a single equation. This approach is supported by the general observation that body shape and proportions are extremely conserved in Ziphiids (Mead et al., 1982). The data and the fitted equation are shown in Figure S1. We assume that 27% of total weight consisted of reserve mass and multiplied the scaling constant by (1 – 0.27) to obtain $\omega_1 = 2.93 \cdot 10^{-5}$. Applying these estimates to newborns that are assumed to receive minimum amount of reserves (Table S2) leads to F_b = 13.9 for *Md* and F_b = 33.2 for *Zc* (Table S3).

Energetic parameters

We set the metabolic rate constant σ_M to 0.6, which equals 2 times the resting metabolic rate as estimated by Kleiber (1975). This low proportionality constant reflects the 'cheap' body structure and low energy expenditure that is associated with extreme deep-diving lifestyle of beaked whales (Pabst et al., 2016). The parameters ε_+ and ε_- represent the anabolic and catabolic conversion efficiencies of build-up and use of reserves. For *Gm*, Hin et al., (2019) argued that the catabolic conversion efficiency resembled the energy density of lipids (reported as 40 MJ kg^{-1} by Lockyer (1993)) and assumed a roughly 90% efficiency of catabolic conversion. This led to the value $\varepsilon_- = 35$. Hin et al. (2019) considered anabolic conversion less efficient and set $\varepsilon_+ = 55$. However, lipids in the blubber of *Gm* are mostly (>90%) triacylglycerols, while blubber lipids of beaked whales are dominated by wax esters (70-100%; Koopman, 2007, 2018). Although wax-rich blubber has a higher energy density than blubber consisting of triacylglycerols, it is currently unknown whether wax esters in the blubber of beaked whales (and other odontocetes) are used as an energy source during periods of energy deficiency (Koopman, 2018). Moreover, the blubber of beaked whales displays low density of microvasculature, suggesting that blubber of beaked whales is relatively inert and mainly serves other functions than that of an energy store (Pabst et al., 2016). If indeed beaked blubber plays a marginal role as energy store,

beaked whales must mobilize other types of lipids or different biochemical compounds (such as proteins) to overcome periods of energy deficiency. To account for the possibility that beaked whales have a lower potential to store energy, we take a precautionary approach and considerably decrease the values of the anabolic and catabolic conversion efficiency parameters compared to *Gm,* and adopt $\varepsilon_{-} = 20$ and $\varepsilon_{+} = 30$ by default.

Table S3: Model parameters

**Symbol**	**Unit**	**Description**	**Md**	**Zc**	**Source**
T_P	day	Gestation period	365	365	1
T_L	day	Lactation period (age at weaning)	986	730	2
T_N	day	Age at which milk consumption starts to decrease	365	365	3
T_R	day	Age at which prey feeding efficiency is 50%	365	365	3
l_b	cm	Structural length at birth	198	270	4, 1
l_∞	cm	Ultimate structural length	475	670	5, 6
k	day^{-1}	Von Bertalanffy growth rate	0.000319	0.000663	this study
ω_1	$kg \cdot cm^{-\omega_2}$	Structural mass-length scaling constant	$2.93 \cdot 10^{-5}$	$2.93 \cdot 10^{-5}$	this study
ω_2	–	Structural mass-length scaling exponent	2.8	2.8	this study
θ_F	–	Relative metabolic cost of reserves	0.2	0.2	7
ρ	–	Target body condition threshold	0.3	0.3	3
ρ_s	–	Starvation body condition threshold	0.15	0.15	3
ϕ_{R_h}	$m^3 \cdot kg^{-2/3} \cdot day^{-1}$	Prey encounter rate high quality	5.0	13.3	8, 9
ϕ_{R_l}	$m^3 \cdot kg^{-2/3} \cdot day^{-1}$	Prey encounter rate low quality	1.0	1.0	this study
ϕ_L	$MJ \cdot kg^{-2/3} \cdot day^{-1}$	Lactation scalar	2.1	2.6	this study
h	$day \cdot kg^{2/3} MJ^{-1}$	Handling time parameter of type II functional response	0.141	0.141	8, 10
η	–	Steepness of assimilation response around target body condition	15	15	3
γ	–	Shape parameter of prey assimilation-age response	2	2	3
ξ_m	–	Non-linearity in female body condition-milk provisioning relation	–2	–2	3
ξ_c	–	Non-linearity in milk assimilation-calve age relation	0.25	0.25	this study

$\boldsymbol{\sigma_M}$	MJ·kg$^{-3/4}$day^{-1}	Field metabolic rate scalar	0.6	0.6	this study
$\boldsymbol{\sigma_G}$	MJ·kg^{-1}	Energetic costs per unit structural mass growth	20	20	this study
$\boldsymbol{\sigma_L}$	–	Lactation conversion efficiency	0.86	0.86	3
$\boldsymbol{\alpha_1}$	day^{-1}	Mortality parameter females & calves	4.01·10^{-4}	4.01·10^{-4}	3
$\boldsymbol{\beta_1}$	day^{-1}	Mortality parameter females & calves	5.82·10^{-4}	5.82·10^{-4}	3
$\boldsymbol{\alpha_2}$	day^{-1}	Mortality parameter females & calves	6.04·10^{-6}	6.04·10^{-6}	3
$\boldsymbol{\beta_2}$	day^{-1}	Mortality parameter females & calves	3.01·10^{-4}	3.01·10^{-4}	3
$\boldsymbol{\mu_{male}}$	day^{-1}	Mortality parameter males	2.13·10^{-4}	2.13·10^{-4}	3
$\boldsymbol{\mu_s}$	day^{-1}	Starvation mortality scalar	0.2	0.2	3
$\boldsymbol{\mu_f}$	day^{-1}	Fetal mortality rate	0.000611	0.000611	this study
$\boldsymbol{\psi}$	day^{-1}	Daily chance of becoming pregnant	0.00225	0.00225	3
$\boldsymbol{\varepsilon_+}$	MJ·kg^{-1}	Anabolic reserves conversion efficiency	30	30	this study
$\boldsymbol{\varepsilon_-}$	MJ·kg^{-1}	Catabolic reserves conversion efficiency	20	20	this study
$\boldsymbol{F_{neo}}$	kg	Amount of reserves required to produce structural mass of neonate	78.98	360	this study
$\boldsymbol{F_b}$	kg	Reserve mass at birth	13.9	33.2	this study
$\boldsymbol{R_{max}}$	MJ·m^{-3}	Max. prey density	0.81	1.35	this study
$\boldsymbol{\delta}$	day^{-1}	Prey turn-over rate	0.05	0.05	3
$\boldsymbol{V}$	m^3	Volume scalar between whale and prey density	1·10^6	1·10^6	3

Sources: (1) Mead (1984); (2) Claridge (2013); (3) Hin et al. (2019); (4) New et al. (2013); (5) MacLeod, (2006); (6) Nishiwaki and Oguro (1972); (7) Pabst et al., (2016); (8) Southall et al. (2019); (9) Benoit-Bird et al., (2020); (10) Tyack et al.(2006).

The parameter σ_G denote the energetics cost of growing one kg of structural mass. For beaked whales, no empirical estimates are available for this parameter, but for *Gm* it was derived to be around 30 MJ kg^{-1} by Hin et al. (2019). In line with the notion that beaked whales are build 'cheap', we deviate from this value and adopt a lower value of $\sigma_G = 20$ MJ kg^{-1}.

The lactation rate scalar parameter ϕ_L was recalculated for *Zc* and *Md* using the approach described in Hin et al. (2019). This approach assumes that milk supply of a female at target body condition ($\frac{F}{W} = \rho_S$) is sufficient to cover metabolic and growth expenses of her calf during

its first year of life. For *Gm* this led to a value of $\phi_L = 2.7$ (Hin et al., 2019) and values for *Md* and *Zc* are similar (Table S3).

The maximum consumption rate of the type II functional response of prey density was estimated based on dive statistics of *Zc*. The average foraging dive cycle (time between start of consecutive deep foraging dives) of *Zc* is on average 121 min (Tyack et al., 2006), which allows *Zc* to perform 11.9 deep dives per day. On each foraging dive there are around 30 prey capture attempts (Tyack et al., 2006), resulting in a maximum consumption of 357 prey items if all attempts are successful. A calorific content of 850 kCal per prey (3.56 MJ; Southall et al., 2019) leads to a maximum consumption rate of 1270 MJ per day. Using this value, the handling time parameter *h* of the type II functional response of a fully-grown *Zc* individual with structural mass of $S_\infty = \omega_1 l_\infty^{\omega_2} = 2398$ kg, becomes $h = 2398^{2/3} / 1270 = 0.141$. We assume that there are no differences in maximum consumption between *Zc* and *Md* other than those related to size. In other words, we do not have any reason to believe that *Zc* and *Md* individuals of identical size have a different maximum consumption rate. With the derived value of $h = 0.141$, the maximum consumption rate of a fully-grown *Md* becomes $\frac{S_\infty^{2/3}}{h} = \frac{915^{2/3}}{0.141} = 669$ MJ day^{-1}.

Pregnancy threshold

The female age at first reproduction is largely determined by the age at which female reserve mass exceeds the pregnancy threshold for the first time (‘age at first receptive’). In addition to the amount of reserves below which individuals experience starvation mortality ($\rho_s W$), the pregnancy threshold includes a constant value (F_{neo}) that can be used to control the modelled age at first receptive. For *Gm*, Hin et al. (2019) assumed that F_{neo} consisted of the amount of stored reserves that are needed to produce a neonate, which can be derived from the structural size at birth ($S_b = \omega_1 l_b^{\omega_2}$) and amounts to:

$$F_{neo} = \frac{\sigma_G\, S_b}{\varepsilon_-} + \frac{\rho_s\, S_b}{1 - \rho_s}$$

Here, the first term represents the amount of female reserve mass required to build the structural mass of a newborn and the second term equals the amount of reserves transferred from the mother to the newborn at birth (F_b). For *Gm* this approach resulted in a modelled age at first reproduction that corresponded well with its observed counterpart (Hin et al., 2021). To infer values of F_{neo} that result in good correspondence between observed age at maturity and the modelled age at first receptive we ran the full population model for a range of F_{neo} – values. We should note that the observed age at maturity for *Md* (9 y) was assumed one year earlier than the observed age at first parturition (Claridge, 2013) and does not include a receptive period before the onset of pregnancy, as is included in our model. Accounting for this 'waiting' period, the modelled age at first receptive should correspond to 9 – 445 / 365 = 7.8 y. For *Zc* we do not know how the estimate for the age at maturity (6.2 y) was derived and the corresponding modelled age at first receptive should therefore be in the range of 5 – 6.2 yrs. The values of F_{neo} reported in Table S3 lead to good correspondence between modelled and observed age at first maturity.

3. Beaked whale satellite telemetry data

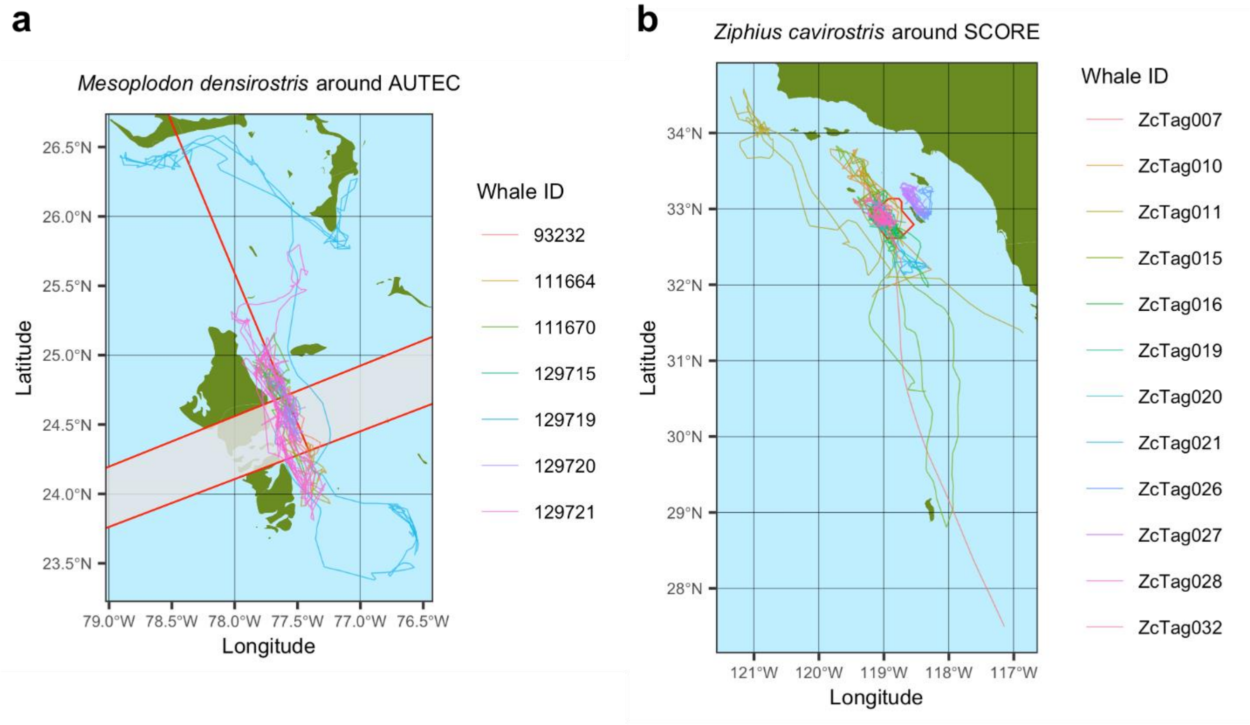

Figure S2: Tracks of a) seven Blainville's beaked whales (*Mesoplodon densirostris*) at AUTEC and b) 12 Cuvier's beaked whales (*Ziphius cavirostris*) at SOAR that were used to estimate transition rate between discrete geographical areas used in the model. Red lines demarcate the boundaries that were used to allocate beaked whale tracks to different model areas. The parts of the tracks that overlap with gray shadings are considered to be on the hydrophone range and all other parts of the tracks (no shading) are off-range areas. In the right panel, these boundaries coincide with the hydrophone array at the Southern California Anti-submarine Warfare Range (SOAR). Bathymetry data were obtained from the GEBCO_2022 Grid (GEBCO Compilation Group, 2022).

4. GAM analysis of hydrophone data

Table S4: Results of the Generalized Additive Model (GAM) on the hydrophone data from AUTEC. Adjusted R-square = 0.142; deviance explained = 9.8%; UBRE = 0.20909; n = 32334

Parametric coefficients	Estimate	Std. error	Z-value	P-value
(Intercept)	-6.291023	0.007530	-835.41	<2e-16
Zone East	-0.350338	0.009431	-37.15	<2e-16
Year 2014	-0.113552	0.009272	-12.25	<2e-16

Smooth terms	Edf	Ref.df	Chi.sq	P-value
Julian day	7.885	8.000	732.004	<2e-16
Hour	7.916	8.000	1178.366	<2e-16
Standardized sonar area	3.197	3.913	89.697	<2e-16
Time since last sonar	7.646	8.522	232.286	<2e-16
Length of sonar event	1.903	2.400	2.906	0.439

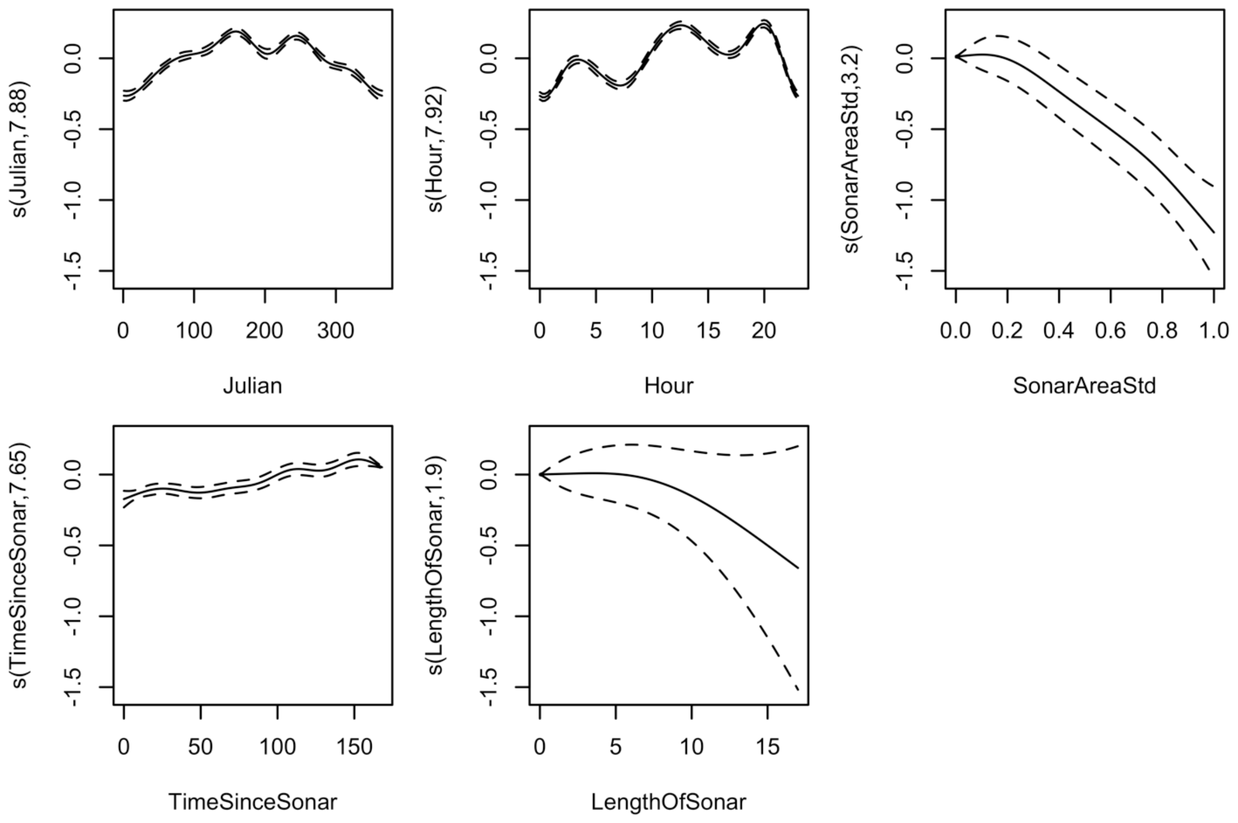

Figure S3: Smooth terms of the GAM on the hydrophone data from AUTEC

Table S5: Results of the Generalized Additive Model (GAM) on the hydrophone data from SOAR. Adjusted R-square = 0.121; deviance explained = 13%; UBRE = 0.24375; n = 23300

Parametric coefficients	Estimate	Std. error	Z-value	P-value
(Intercept)	-20.80495	0.01197	-1738.60	<2e-16
Zone East	-0.21907	0.01331	-16.45	<2e-16
Year 2015	-0.20165	0.01359	-14.83	<2e-16

Smooth terms	Edf	Ref.df	Chi.sq	P-value
Julian day	7.841	8.000	1882.26	<2e-16
Hour	6.399	8.000	970.94	<2e-16
Standardized sonar area	1.596	1.975	76.54	<2e-16
Time since last sonar	6.282	7.363	118.69	<2e-16
Length of sonar event	1.005	1.010	10.61	0.00111

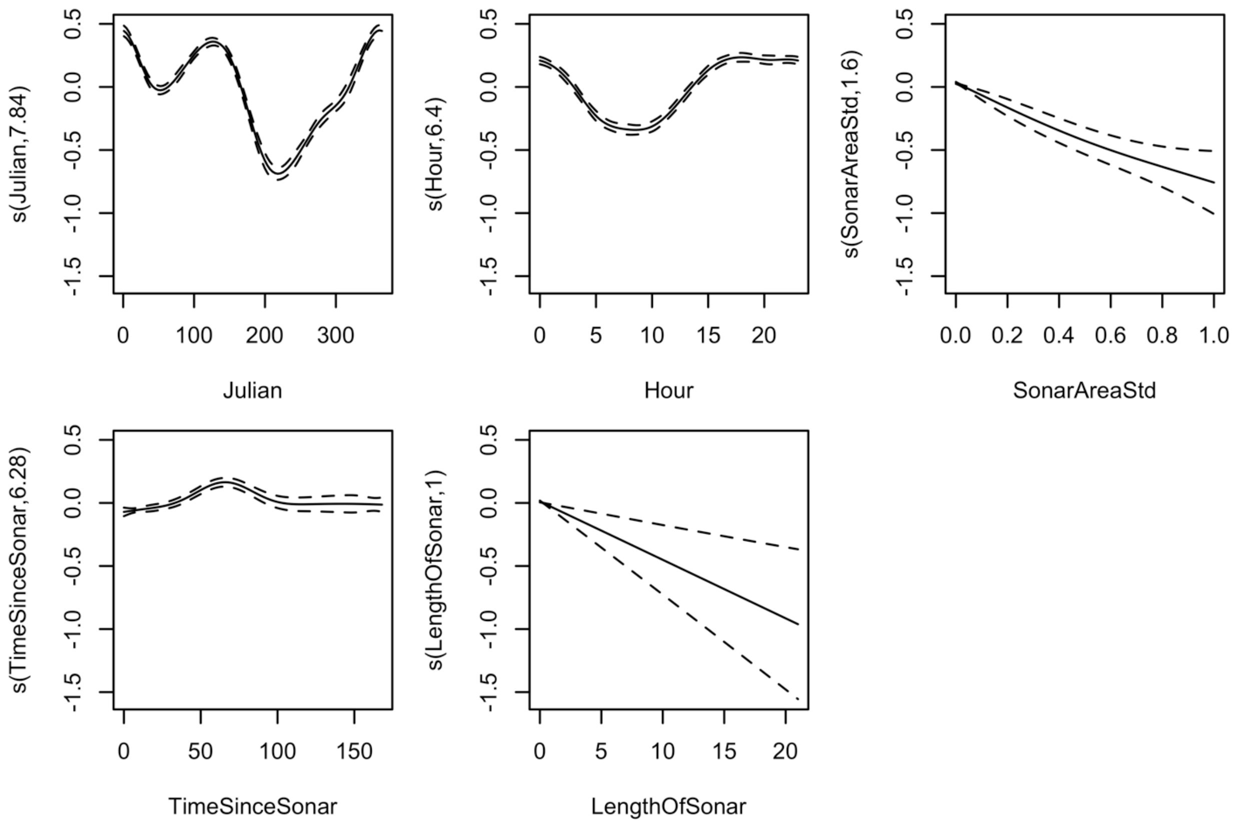

Figure S4: Smooth terms of the GAM on the hydrophone data from SOAR

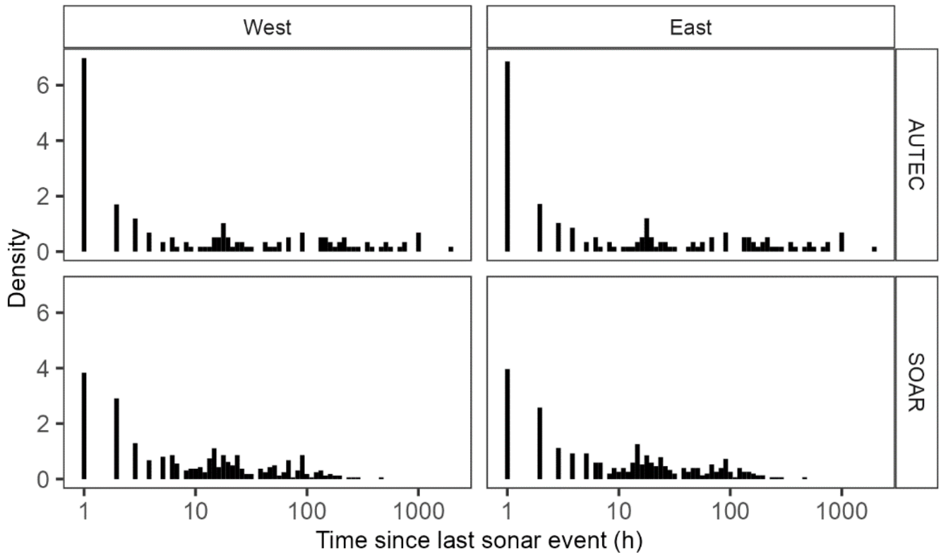

Figure S5: Distribution of the time since last MFAS use at AUTEC and SOAR ranges, showing both western and eastern range areas.

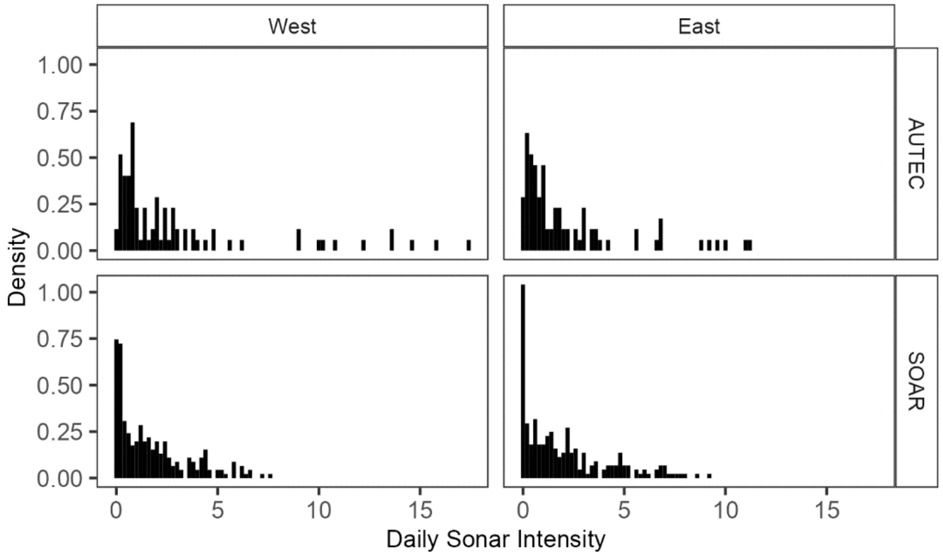

Figure S6: Distribution of MFAS use aggregated by day (daily sum of standardized sonar area) for both western and eastern areas of the AUTEC and SOAR ranges

5. Supplemental Results

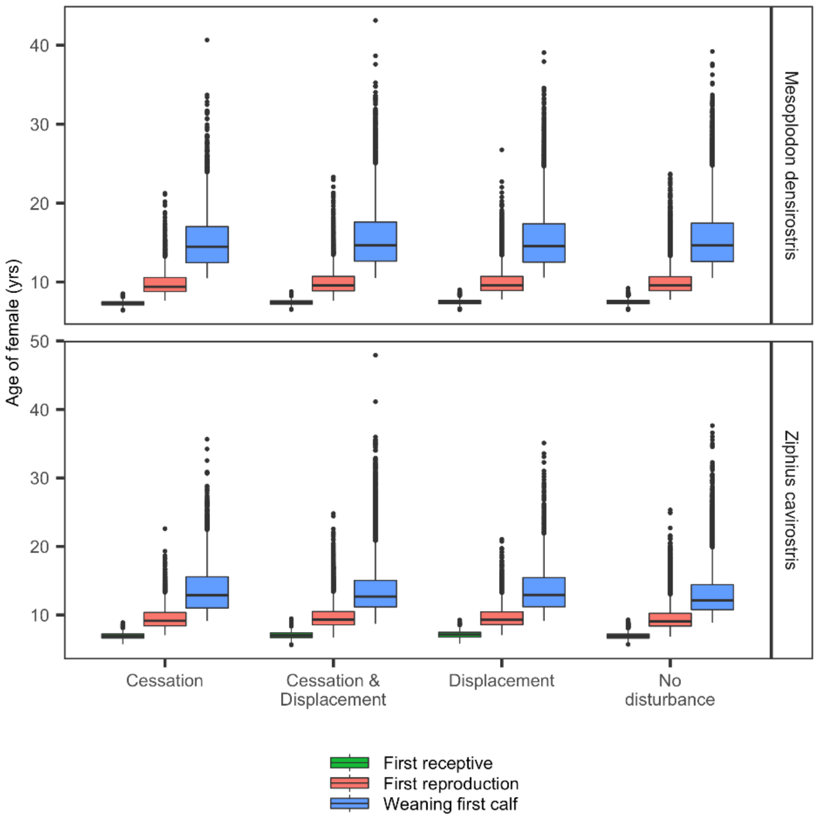

Figure S7: Distributions of female age at onset of reproduction for the different behavioral response scenarios, distinguishing between age at first receptive, age at first reproduction and female age at weaning first calf. Data represent ages of females from a stationary population. For *Zc* a R_{max} – value of 2 was used for the cessation and displacement scenario, as the population went extinct with the default value of R_{max} = 1.35. Boxplots show medians (horizontal bars) and 25% and 75% quantiles (box edges). Whiskers extend 1.5 times the inter quartile range beyond the edges of the box, or indicate extreme values if these fall within this range.

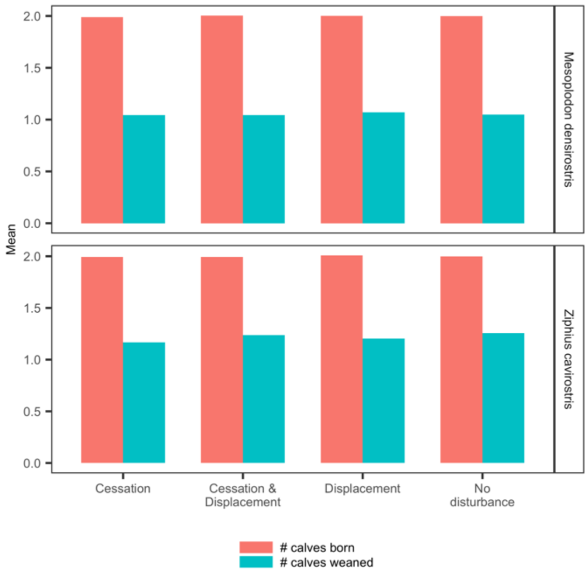

Figure S8: Lifetime reproductive output in terms of the mean number of calves born and weaned during the entire life of a single female. Bars represent mean values from all females living in stationary populations under a certain disturbance regime.

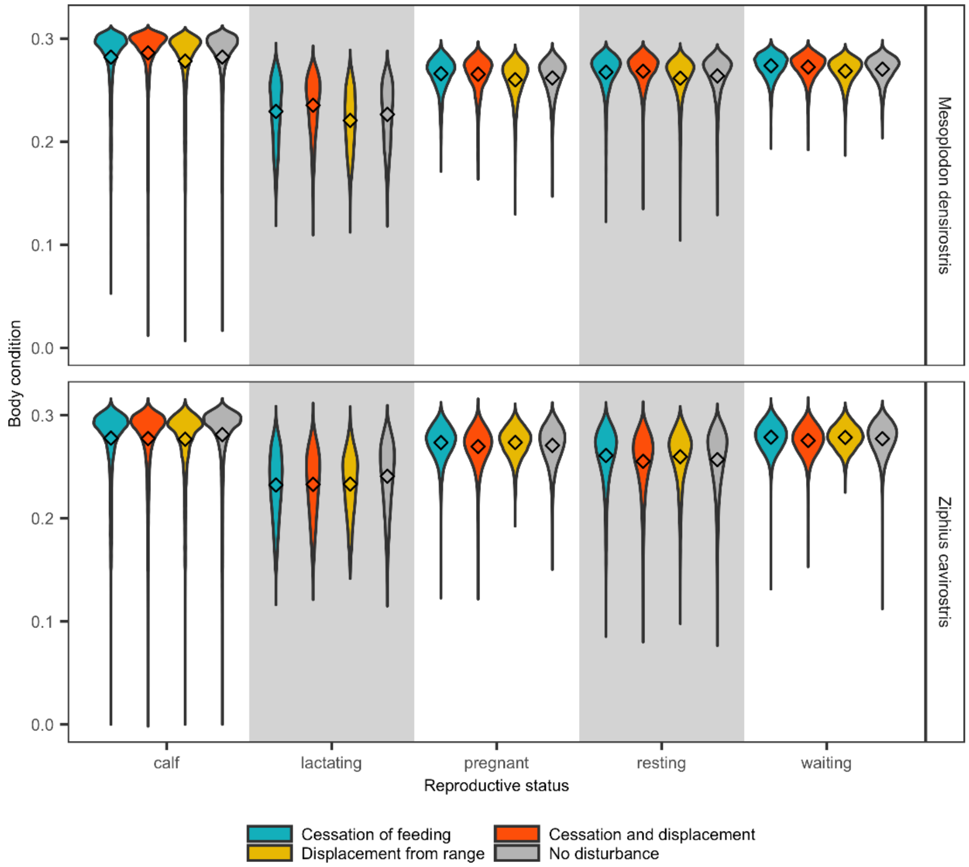

Figure S9: Distribution of female body condition as a function of reproductive status with and without disturbance from MFAS. Triangles indicate mean values and shapes indicate the distribution and total range of the data. Females with body condition below the starvation body condition threshold of 0.15 suffered from increased mortality. The lactating female group also represent females that are simultaneously ‘waiting’ or ‘pregnant’. Data represent body condition values of all females in a stationary population from single simulation of 100 y, with output collected each day. For *Zc* a R_{max} – value of 2 was used for the cessation of displacement scenario, as the population went extinct with the default value of R_{max} = 1.35.

Table S6: Mean (sd) of several life history variables and body condition (BC) of modelled *Md* and *Zc* females.

	Mesoplodon densirostris		*Ziphius cavirostris*	
	Undisturbed	*Disturbed*[*]	*Undisturbed*	*Disturbed*[*]
Age at first receptive	7.5 (0.31)	7.3 (0.29)	6.93 (0.45)	6.94 (0.45)
Age at first reproduction	10 (1.66)	9.9 (1.67)	9.55 (1.71)	9.61 (1.72)
Age at weaning first calf	15.5 (3.79)	15.3 (3.77)	13.09 (3.16)	13.79 (3.56)
Number of calves born	2.00 (3.00)	1.99 (2.97)	2.00 (3.19)	1.99 (3.10)
Number of calves weaned	1.05 (1.83)	1.04 (1.81)	1.26 (2.20)	1.17 (2.03)
BC calves	0.282 (0.0306)	0.286 (0.0282)	0.281 (0.0316)	0.277 (0.0325)
BC resting females	0.264 (0.0203)	0.268 (0.0175)	0.257 (0.0299)	0.255 (0.0282)
BC waiting females	0.270 (0.0123)	0.272 (0.0124)	0.277 (0.0156)	0.275 (0.0141)
BC pregnant females	0.262 (0.0179)	0.265 (0.0163)	0.271 (0.0189)	0.270 (0.0174)
BC lactating females	0.226 (0.0295)	0.235 (0.0277)	0.241 (0.0325)	0.233 (0.0319)

[*]Disturbance leads to cessation of foraging

6. References

Barlow, J., Boveng, P., 1991. Modelling age-specific mortality for marine mammal populations. Mar Mamm Sci 7, 50–65. https://doi.org/10.1111/j.1748-7692.1991.tb00550.x

Benoit-Bird, K., Southall, B., Moline, M., Claridge, D., Dunn, C., Dolan, K., Moretti, D., 2020. Critical threshold identified in the functional relationship between beaked whales and their prey. Mar. Ecol. Prog. Ser. https://doi.org/10.3354/meps13521

Claridge, D.E., 2013. Population ecology of Blainville's beaked whales (Mesoplodon densirostris). University of St. Andrews, St. Andrews, United Kingdom.

De Roos, A.M., Galic, N., Heesterbeek, H., 2009. How resource competition shapes individual life history for nonplastic growth: ungulates in seasonal food environments. Ecology 90, 945–960. https://doi.org/10.1890/07-1153.1

GEBCO Compilation Group, 2022. GEBCO 2022 Grid. https://doi.org/10.5285/e0f0bb80-ab44-2739-e053-6c86abc0289c

Heyning, J.E., 1989. Cuvier's beaked whale Ziphius cavirostris G. Cuvier, 1823, in: Ridgway, S.H., Harrison, R. (Eds.), Handbook of Marine Mammals. London and San Diego, pp. 289–308.

Hin, V., Harwood, J., de Roos, A.M., 2021. Density dependence can obscure nonlethal effects of disturbance on life history of medium-sized cetaceans. PLoS ONE 16, e0252677. https://doi.org/10.1371/journal.pone.0252677

Hin, V., Harwood, J., De Roos, A.M., 2019. Bio-energetic modeling of medium-sized cetaceans shows high sensitivity to disturbance in seasons of low resource supply. Ecol Appl 29. https://doi.org/10.1002/eap.1903

Kleiber, M., 1975. The fire of life: an introduction to animal energetics. R.E. Krieger Pub. Co., Huntington, N.Y.

Kooijman, B., 2009. Dynamic energy budget theory for metabolic organisation, 3rd ed. Cambridge University Press, Cambridge. https://doi.org/10.1017/CBO9780511805400

Koopman, H.N., 2018. Function and evolution of specialized endogenous lipids in toothed whales. J Exp Biol 221, jeb161471. https://doi.org/10.1242/jeb.161471

Koopman, H.N., 2007. Phylogenetic, ecological, and ontogenetic factors influencing the biochemical structure of the blubber of odontocetes. Mar Biol 151, 277–291. https://doi.org/10.1007/s00227-006-0489-8

Lockyer, C., 2007. All creatures great and smaller: a study in cetacean life history energetics. J. Mar. Biol. Ass. 87, 1035–1045. https://doi.org/10.1017/S0025315407054720

Lockyer, C., 1993. Seasonal changes in body fat condition of Northeast Atlantic pilot whales, and their biological significance. Rep Int Whal Commn (Special Issue 14) 325–350.

MacLeod, C.D., 2006. How big is a beaked whale? A review of body length and sexual size dimorphism in the family Ziphiidae. J Cetacean Res Manage 7, 301–308.

Mead, J.G., 1984. Survey of Reproductive Data for the Beaked Whales (*Ziphiidae*). Rep Int Whal Commn (Special Issue 6) 91–96.

Mead, J.G., Walker, W.A., Houck, W.J., 1982. Biological Observations on Mesoplodon carlhubbsi (Cetacea: Ziphiidae), Smithsonian Contributions to Zoology. Smithsonian Institution Press, Washington.

New, L.F., Moretti, D.J., Hooker, S.K., Costa, D.P., Simmons, S.E., 2013. Using energetic models to investigate the survival and reproduction of beaked whales (family *Ziphiidae*). PLoS ONE 8, e68725. https://doi.org/10.1371/journal.pone.0068725

Nishiwaki, M., Oguro, N., 1972. Catch of the Cuvier's Beaked Whales Off Japan in Recent Years. Sci. Rep. Whales Res. Inst. 35–41.

Pabst, D.A., McLellan, W.A., Rommel, S.A., 2016. How to build a deep diver: the extreme morphology of Mesoplodonts. Integr. Comp. Biol. 56, 1337–1348. https://doi.org/10.1093/icb/icw126

Siler, W., 1979. A competing-risk model for animal mortality. Ecology 60, 750–757. https://doi.org/10.2307/1936612

Southall, B.L., Benoit-Bird, K.J., Moline, M.A., Moretti, D., 2019. Quantifying deep-sea predator–prey dynamics: Implications of biological heterogeneity for beaked whale conservation. J Appl Ecol 56, 1040–1049. https://doi.org/10.1111/1365-2664.13334

Tyack, P.L., Johnson, M., Soto, N.A., Sturlese, A., Madsen, P.T., 2006. Extreme diving of beaked whales. Journal of Experimental Biology 209, 4238–4253. https://doi.org/10.1242/jeb.02505
